# JNK1 and ERK1/2 modulate lymphocyte homeostasis via BIM and DRP1 upon AICD induction

**DOI:** 10.1038/s41418-020-0540-1

**Published:** 2020-04-28

**Authors:** Luca Simula, Mauro Corrado, Benedetta Accordi, Anthea Di Rita, Francesca Nazio, Ylenia Antonucci, Arianna Di Daniele, Federico Caicci, Ignazio Caruana, Maria Eugenia Soriano, Martina Pigazzi, Franco Locatelli, Francesco Cecconi, Silvia Campello

**Affiliations:** 1 Department of Biology, University of Rome Tor Vergata, Rome, Italy; 2 Venetian Institute of Molecular Medicine, Padua, Italy; 3 Department of Woman and Child Health, University of Padua, Padua, Italy; 4 IRCCS Santa Lucia Foundation, Rome, Italy; 5 Department of Pediatric Hemato-Oncology and Cell and Gene Therapy, IRCCS Bambino Gesù Children Hospital, Rome, Italy; 6 Department of Biology, University of Padua, Padua, Italy; 7 Department of Pediatrics, University of Rome La Sapienza, Rome, Italy; 8 Unit of Cell Stress and Survival, Danish Cancer Society Research Center, Copenhagen, Denmark; 9 Present Address: Max Planck Institute of Immunology and Epigenetics, Freiburg im Breisgau, Germany

**Keywords:** Cell biology, Immune cell death, Preclinical research

## Abstract

The Activation-Induced Cell Death (AICD) is a stimulation-dependent form of apoptosis used by the organism to shutdown T-cell response once the source of inflammation has been eliminated, while allowing the generation of immune memory. AICD is thought to progress through the activation of the extrinsic Fas/FasL pathway of cell death, leading to cytochrome-C release through caspase-8 and Bid activation. We recently described that, early upon AICD induction, mitochondria undergo structural alterations, which are required to promote cytochrome-C release and execute cell death. Here, we found that such alterations do not depend on the Fas/FasL pathway, which is instead only lately activated to amplify the cell death cascade. Instead, such alterations are primarily dependent on the MAPK proteins JNK1 and ERK1/2, which, in turn, regulate the activity of the pro-fission protein Drp1 and the pro-apoptotic factor Bim. The latter regulates *cristae* disassembly and cooperate with Drp1 to mediate the Mitochondrial Outer Membrane Permeabilization (MOMP), leading to cytochrome-C release. Interestingly, we found that Bim is also downregulated in T-cell Acute Lymphoblastic Leukemia (T-ALL) cells, this alteration favouring their escape from AICD-mediated control.

## Introduction

FasL interaction with Fas receptors promotes activating-cleavage of caspase-8 and Bid, leading to activation of the mitochondrial pathway of apoptosis, characterized by cytochrome-C (cyt-C) release from mitochondria and cell death [1]. This Fas/FasL pathway plays a key role during a specialized form of controlled cell death in T cells, following T-cell receptor (TCR) stimulation, and known as Activation-Induced Cell Death (AICD) [2]. AICD is crucial to shutdown T-cell response once target cells have been eliminated, thus avoiding the generation of autoimmunity-driving, chronically active T cells [3]. Also, excessive AICD is one of the major obstacles for anti-cancer immuno-therapies, characterized by a too short lifespan of infused T cells [4]. On the other side, resistance to AICD is observed in leukemic T cells, being partially responsible for their survival and progression [5]. Besides Fas/FasL pathway, additional pathways have been proposed to regulate AICD in T cells [6], such as Mitogen-Activated Protein Kinase (MAPK) pathway [7]. Although it regulates several apoptosis-related proteins, such as Drp1, Bim and Nur77 [8–11], how and when MAPK pathway is playing a role during AICD is still not known.

We recently described that AICD progression requires dramatic alterations of mitochondria morphology to allow cyt-C release in the cytosol [12]. After TCR engagement, mitochondria fragment, loose their membrane potential, and disassembly their *cristae* (where cyt-C is normally stored), all hallmarks of the mitochondrial apoptotic pathway [13, 14]. Since autophagy is meanwhile inhibited in AICD, such damaged mitochondria cannot be removed through autophagy, this leading to cell death [12]. While the molecular pathway responsible for autophagy inhibition has been well described [12], the molecular regulators of such mitochondria alterations are less characterized. We previously showed a role for calcium/calcineurin-dependent regulation of Drp1, as well as of Opa-1 cleavage, during initial stages of AICD. Nevertheless, it is still unknown their temporal relationship with the Fas/FasL pathway, i.e., if they precede or follow its activation. The same can be said about the involvement of Bcl-2 family members, which are additional important regulators of AICD progression [15, 16]. Thus, dissecting the molecular regulation of these events would be extremely helpful to propose new therapeutic strategies in pathological conditions, such as autoimmunity and cancer.

 We here found that the early steps of AICD induction are exclusively characterized by mitochondria alterations, while the classical Fas/FasL pathway is instead required in a second, late phase to amplify the apoptotic cascade. Moreover, we found that MAPK proteins c-Jun N-terminal Kinase 1 (JNK1) and Extracellular-Regulated Protein Kinases 1/2 (ERK1/2) control mitochondria alterations early upon TCR engagement during AICD, by modulating two key pro-apoptotic proteins, the Bcl-2 family member Bim, and the mitochondrial pro-fission protein Drp1. Last, in a readout of the highest biomedical importance, we also observed that Bim is downregulated in T-cell Acute Lymphoblastic Leukemia (T-ALL) primary cells, this favouring their escape from the AICD-mediated control.

## Results

### The Fas/FasL apoptotic pathway is involved only late in AICD progression

**Fig. 1:**
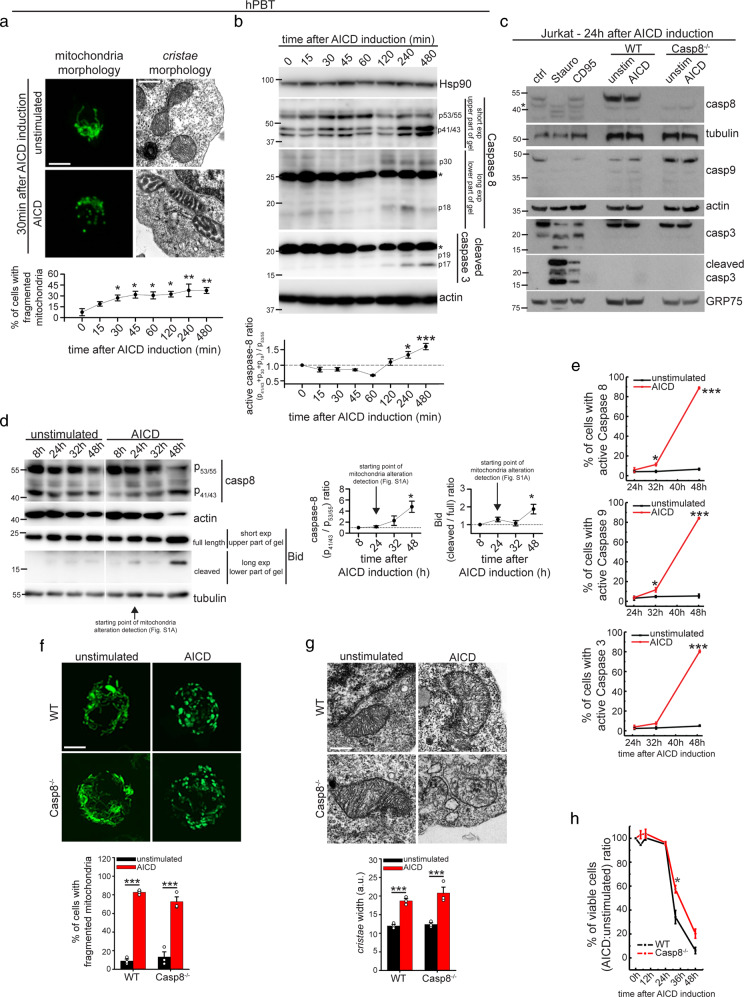
Caspase-8-dependent extrinsic cell death pathway is not involved in early AICD events. **a** hPBT cells representative z-stacks reconstructions of TOM20 staining (left panel) and representative electron micrographs (right panel), 30 min after AICD induction. Quantification of the cells with fragmented mitochondria at the indicated time after AICD induction is reported in the graph below (*n* = 3). **b** Expression levels of the indicated proteins evaluated by western blot in hPBT cells at the indicated times after AICD induction. Relative quantification of the active caspase-8 (measured as the ratio between active p41/43, p30 and p18 isoforms compared with inactive p53/55 isoform) is reported in the graph below (*n* = 3). Asterisks (*) indicate unspecific bands. **c** Expression levels of the indicated proteins evaluated by western blot in WT and caspase-8^−/−^ Jurkat cells 24 h after AICD induction, i.e., the starting point of mitochondria alteration detection, as shown in Fig. S1A (one experiment representative of three independent experiments). **d** Expression levels of the indicated proteins evaluated by western blot in Jurkat cells at the indicated time points after AICD induction. Relative quantifications of the active caspase-8 (measured as the ratio between active p41/43 compared with inactive p53/55 isoform) and active Bid (measured as cleaved/full length ratio) are reported in the graphs on the right (caspase-8: *n* = 3; Bid: *n* = 4). **e** Percentage of control or AICD-stimulated Jurkat cells with active caspases, as assessed by CaspGlow kit by flow cytometry at the indicated times after AICD induction (*n* = 3). **f** Representative reconstructions of z-stacks of the transiently transfected mtYFP fluorescence 24 h after AICD induction in WT and caspase-8^−/−^ Jurkat cells. Quantification of the percentage of cells with fragmented mitochondria is reported in the graph (*n* = 3). **g** Representative electron micrographs of WT and caspase-8^−/−^ Jurkat cells 24 h after AICD induction. Quantification of the maximum *cristae* width in each condition is reported in the graph (*n* = 3). **h** Relative viability (AICD:unstimulated cells ratio) of WT and caspase-8^−/−^ Jurkat cells stimulated for AICD (percentage of annexinV^negative^ cells assessed by flow cytometry) (*n* = 3). Data are shown as mean ± SEM. Scale bar, 10 μm in **a** (IF), 0.5 μm in **a** (TEM), 10 μm in **f**, and 0.35 μm in **g**. Significance is indicated as follows: *p < 0.05; ***p < 0.001.

Mitochondria fragmentation and *cristae* widening occur as early as 30 min after AICD induction in hPBT cells and 24 h after AICD induction in Jurkat cells (Fig. 1a and S1A), a time point when apoptosis is not observed yet [12]. Interestingly, caspase-3, caspase-9 and FasL/Fas pathway-dependent caspase-8 are not cleaved and active at this time point, but only later (Fig. 1b–e). In line with this, cleaved forms of Bid, a caspase-8 target, are observed only at later time points in Jurkat cells, also consistent with the timing of caspase-8 activation (Fig. 1d). For further verifying the requirement of FasL/Fas and caspase-8 involvement in mitochondria alterations during AICD, we took advantage of caspase-8 KO Jurkat cells (Fig. S1B), which are protected from CD95-mediated, but not staurosporine-mediated cleavage of Bid and cell death (Fig. S1C). Interestingly, caspase-8 KO Jurkat cells normally fragment mitochondria and disassembly their *cristae* upon AICD induction (Fig. 1f, g). Further confirming that caspases are not involved in mitochondria structural alterations, pan-caspase inhibitor zVAD-FMK does not prevent Opa-1 oligomers cleavage, mitochondria fragmentation, mitochondria membrane potential (MMP) depolarization and *cristae* disassembly in AICD-induced Jurkat cells (Fig. S1D–G). By contrast, zVAD-FMK efficiently prevents etoposide-dependent apoptosis and Bid cleavage in Jurkat cells, as expected (Fig. S1H–I). Also, caspase-8 KO Jurkat cells are protected from cell death during AICD only at later time points (Fig. 1h), similarly to FAS-insensitive Jurkat cells (Fig. S1J), and in line with the timing of caspase-8 activation.

Therefore, the caspase-8-mediated FasL/Fas pathway is dispensable for mitochondria structural alterations during AICD induction, and is involved only at later time points, probably in the amplification of the cell death cascade.

### Pharmacological targeting of MAPK pathway prevents early events upon AICD induction

Next, we asked which signaling pathways could account for the mitochondria alterations during AICD. Since MAPK pathway promotes FasL upregulation following T-cell stimulation [17], it is believed to play its pro-apoptotic role mainly through FasL/Fas pathway modulation. However, MAPK proteins can also directly modulate Bim and Drp1 [8, 18], two important regulators of the mitochondrial apoptotic pathway [13, 14]. Furthermore, drugs targeting the MAPK pathway have been reported to prevent T-cell death [7]. These considerations prompted us to investigate whether the MAPK pathway regulates mitochondria alterations also during AICD, independently from FasL/Fas. We tested AICD induction in hPBT cells in the presence of two different MAPK protein inhibitors: SP600125 (ideally more specific for JNK) and FR180204 (ideally more specific for ERK).

**Fig. 2:**
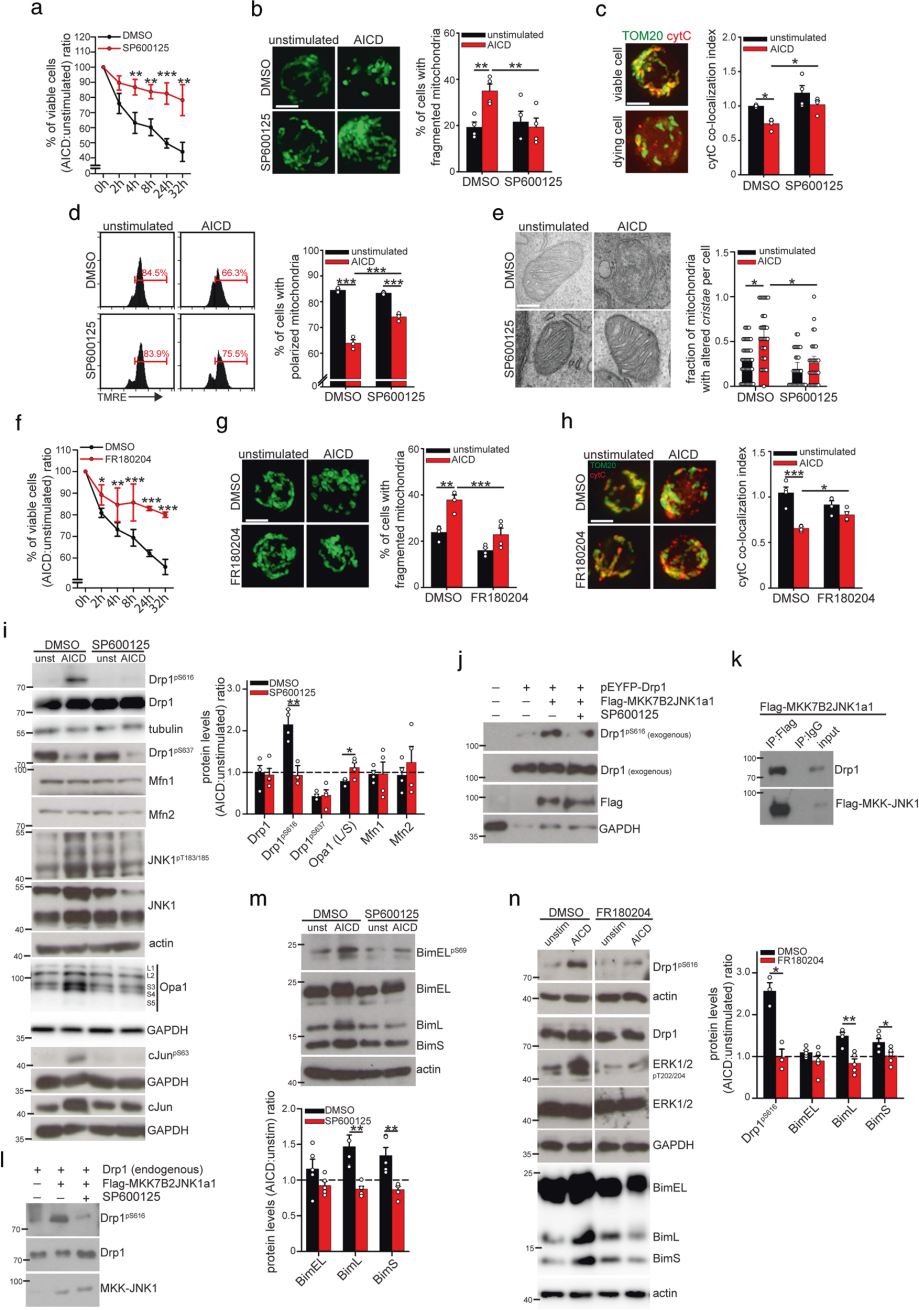
Treatment with SP600125 and FR180204 inhibitors prevents mitochondrial alterations upon AICD in hPBT cells. **a–e** hPBT cells have been stimulated for AICD induction in presence or not of SP600125. In **a** is reported the relative viability (AICD:unstimulated ratio) assessed by flow cytometry (percentage of annexinV^negative^7AAD^negative^ cells assessed by flow cytometry) (*n* = 5). In **b** are reported representative confocal z-stack reconstructions of mitochondria (TOM20) in hPBT cells 2 h after AICD induction (*n* = 4). In **c** are reported representative images of viable and dying hPBT cells showing mitochondria (TOM20 in green, confocal z-stack reconstruction) and cytochrome-C (red) localization, 4 h after AICD induction. Quantification of the cyt-C co-localization index with mitochondria (see Methods for details) in presence or not of SP600125 4 h after AICD induction is reported in the graph on the right (*n* = 4). In **d** is reported the TMRE profile by flow cytometry 2 h after AICD induction. Percentages of cells with polarized mitochondria (indicated with red bars on plots) are quantified in the graph on the right (*n* = 3). In **e** are reported representative electron micrographs of hPBT cells 4 h after AICD induction. Quantification of the fraction of mitochondria with altered *cristae* per cell (range 0–1) in each condition is reported in the graph on the right (at least 30 cells per condition from *n* = 3 independent experiments). **f** Relative viability (AICD:unstimulated ratio) of hPBT cells stimulated for AICD induction in presence or not of FR180204 (percentage of annexinV^negative^7AAD^negative^ cells assessed by flow cytometry) (*n* = 4). **g** Representative confocal z-stack reconstructions of mitochondria (TOM20) in hPBT cells 2 h after AICD induction in presence or not of FR180204. Quantification of the cells with fragmented mitochondria is reported in the graph on the right (*n* = 4). **h** Representative images showing mitochondria (TOM20 in green, confocal z-stack reconstructions) and cytochrome-C (red) localization 4 h after AICD induction in hPBT cells, in presence or not of FR180204. Quantification of the cytochrome-C co-localization index with mitochondria (see Methods for details) is reported in the graph on the right (*n* = 4). **i** Expression levels of the indicated (phospho)-proteins in hPBT cells 2 h after AICD induction, in presence or not of SP600125. Relative quantification of the AICD:unstimulated ratio for each protein is reported in the graph on the right (*n* = 4). **j** Jurkat cells have been transfected with pEYFP-C1-Drp1 and/or Flag-MKK7B2JNK1a1 plasmids. After 18 h, cells were incubated or not in presence of SP600125 for 6 h and then analyzed for the expression levels of the indicated (phospho)-proteins (one experiment representative of three independent experiments). **k, l** HeLa cells were transfected or not with Flag-MKK7B2JNK1a1 plasmid. After 24 h, cells were lysed and protein extract immunoprecipitated (IP) using anti-Drp1, anti-Flag or control IgG antibody. In **k** is reported the co-immunoprecipitation between Drp1 and MKK7B2JNK1a1 in anti-Flag immuno-precipitated lysates. In **l** an in vitro kinase assay has been performed with IP-purified endogenous Drp1 (from untransfected HeLa) and Flag-MKK7B2JNK1a1 (from HeLa cells transfected with Flag-MKK7B2JNK1a1) in presence or not of SP600125 for 30 min; then the levels of the indicated (phospho)-proteins has been measured by western blot. All experiments are representative of three independent experiments. **m** Expression levels of the indicated (phospho)-proteins in hPBT cells 2 h after AICD induction in presence or not of SP600125. Relative quantification of the AICD:unstimulated ratio for each Bim isoform is reported in the graph below (*n* = 5). **n** Expression levels of the indicated (phospho)-proteins in hPBT cells 2 h after AICD induction in presence or not of FR180204. Relative quantification of the AICD:unstimulated ratio for each protein is reported in the graph on the right (Drp1: *n* = 3; Bim *n* = 5). Data are shown as mean ± SEM. Scale bar, 5 μm in **b, c, g, h**, 0.15 μm in **e**. Significance is indicated as follows: *p < 0.05; **p < 0.01; ***p < 0.001.

SP600125 not only efficiently prevents primary hPBT cell death following AICD induction (Fig. 2a), but it prevents all the classical mitochondria structural alterations observed during AICD: network fragmentation (Fig. 2b), cyt-C release (Fig. 2c and S2A), mitochondria membrane potential depolarization (Fig. 2d), and *cristae*-remodeling (Fig. 2e). In addition, Jurkat cells expressing a dominant-negative JNK protein show reduced cell death upon AICD (Fig. S2B) and do not fragment their network 24 h after AICD induction (Fig. S2C). Similarly, treatment of primary hPBT cells with the ERK1/2 inhibitor FR180204 prevents cell death (Fig. 2f), mitochondria fragmentation (Fig. 2g) and cyt-C release (Fig. 2h and S2A) upon AICD. When looking at the mitochondria-shaping proteins, we observed that SP600125 treatment reduces Opa-1 conversion from long to short forms (consistent with the inhibition of *cristae* disassembly) and Drp1-activating phosphorylation on Ser616 (Fig. 2i), while no effect is observed in the regulation of Drp1 Ser637 phosphorylation, which is a known target of the calcium/calcineurin pathway [19].

Computational analysis on Group-Based Prediction System indicates that JNK could bind and phosphorylate both human and murine Drp1 on Ser616 (isoform 1, peptide PIPIMPASPQKGHAV, score 3.699, cut-off 3.02). Therefore, we decided to investigate this putative interaction. First, we proved that Drp1 phosphorylation also occurs in presence of actinomycin-D, similarly to c-Jun one (Fig. S2D), excluding that JNK1 mediates this event through its classical activity as inducer of transcription factors. Second, overexpression of an active form of JNK1 (Flag-MKK7B2JNK1a1) leads to Drp1 phosphorylation on Ser616 in Jurkat cells (assessed on cells over-expressing YFP-Drp1), and this is prevented by SP600125 treatment (Fig. 2j). Third, we co-immunoprecipitated Drp1 and MKK7B2JNK1a1 in HeLa cells, confirming their interaction (Fig. 2k). Last, through an in vitro kinase assay with IP-purified Flag-MKK7B2JNK1a1 and endogenous Drp1, we found that JNK1 is able to phosphorylate Drp1 on Ser616 and that SP600125 prevents this event (Fig. 2l).

The pro-apoptotic protein Bim regulates apoptosis in T cells [20] and is a known regulator of the mitochondrial pathway of cell death [21]. Consistently, we found that both Bim-L and -S isoforms are upregulated upon AICD induction, but SP600125 treatment prevents such modulation (Fig. 2m). By contrast, the longest Bim-EL isoform is not upregulated upon AICD (Fig. 2m), due to its MAPK protein-dependent [11] phosphorylation on Ser69 (Fig. 2m), which mediates its proteasomal degradation (Fig. S2E).

Last, Drp1 phosphorylation on Ser616 and upregulation of shorter Bim-L and -S isoforms do not occur in presence of ERK inhibitor FR180204, too (Fig. 2n).

Of note, Drp1 is already modestly accumulated on mitochondria in unstimulated hPBT cells, and it does not accumulate further upon AICD induction (Fig. S2A). Although Drp1 is dephosphorylated on Ser637 during AICD (Fig. 2i), our data are consistent with a recent report indicating that the phosphorylation status on Ser637 may rather control Drp1 activity than its translocation to mitochondria [22]. Also, AICD stimulation promotes phosphorylation of both cytosolic and mitochondrial pools of Drp1 on Ser616, and SP600125 and FR180204 (two MAPK inhibitors) prevent such phosphorylation (Fig. S2A). This suggests that, during AICD, MAPK proteins are required to activate the mitochondrial pool of Drp1 through phosphorylation on its residue Ser616 to allow mitochondria fragmentation, and then trigger cell death.

In sum, pharmacological inhibitors of the MAPK pathway prevent the mitochondria structural alterations observed upon AICD induction in T cells, thus showing a new role for JNK and ERK on regulating AICD.

### JNK1 and ERK1/2 play distinct roles during AICD induction

**Fig. 3:**
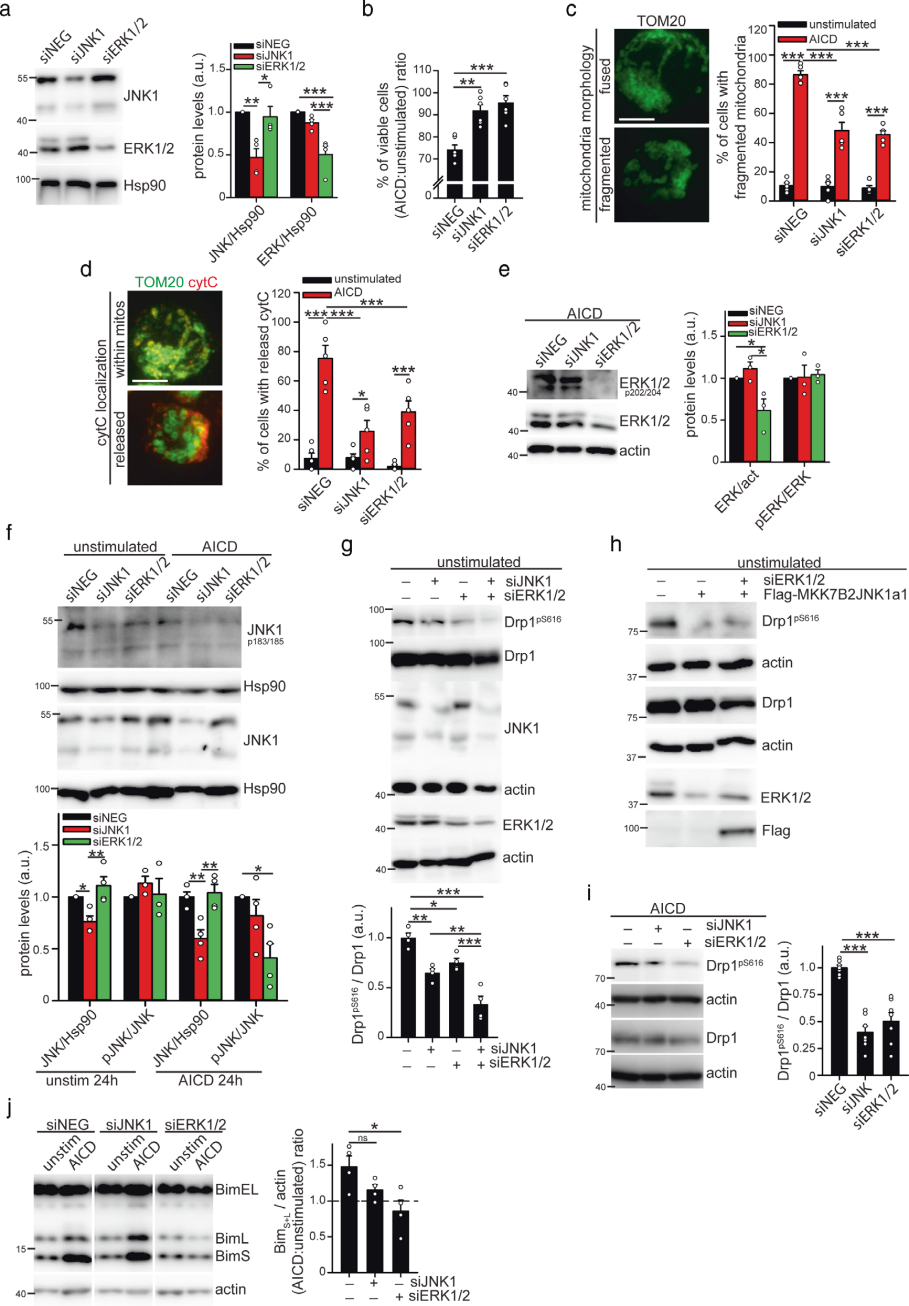
Dissection of JNK1 and ERK1/2 roles during AICD progression. Jurkat cells have been transfected with the indicated siRNA or with Flag-MKK7B2JNK1a1 plasmid (only in panel **h**). 24 h after transfection, Jurkat cells have been simulated for AICD. **a** Representative western blot showing transfection efficiency in Jurkat cells (*n* = 4). **b** Relative viability (AICD:unstimulated ratio) of transfected Jurkat cells stimulated for AICD induction (percentage of annexinV^neg^7AAD^neg^ cells assessed by flow cytometry) (*n* = 6). **c** On the left: representative images of mitochondria morphology (TOM20, confocal z-stack reconstructions) 24 h after AICD induction in Jurkat cells transfected with siNEG. On the right: quantification of the percentage of Jurkat cells with fragmented mitochondria upon AICD induction and after transfection with the indicated siRNAs (*n* = 5). **d** Representative images of viable and dying Jurkat cells transfected with siNEG showing mitochondria (TOM20 in green, confocal z-stack reconstructions) and cytochrome-C (red) localization 28 h after AICD induction. Quantification of the percentage of cells with cyt-C released from mitochondria upon AICD induction and after transfection with the indicated siRNAs is reported in the graph on the right (*n* = 5). **e–j** Expression levels of the indicated (phospho)-proteins in transfected Jurkat cells. In **e**, **f**, **i**, **j** cells have been analyzed 24 h after AICD induction. In **g**, **h** cells have been analyzed 24 h after transfection without AICD induction (unstimulated). Quantifications of the expression level of the relative proteins are reported in the corresponding graphs (**e**: *n* = 3; **f**: JNK *n* = 4, pJNK *n* = 3 unstim, pJNK *n* = 4 AICD; **g**–**j**: *n* = 4; **h** is one experiment representative of three independent experiments; **i**: *n* = 7). Data are shown as mean ± SEM. Scale bar, 10 μm in **c** and **d**. Significance is indicated as follows: *p < 0.05; **p < 0.01; ***p < 0.001; ns not significant.

Pharmacological inhibitors of MAPK proteins frequently show off-target effects [23], and a limited specificity toward single MAPK proteins at the concentrations frequently used. Therefore, to better distinguish the exact role played by JNK1 and ERK1/2 kinases during AICD, we decided to down-regulate their expression in Jurkat cells by siRNAs (Fig. 3a). Silencing of either JNK1 or ERK1/2 increases cell survival following AICD induction (Fig. 3b), prevents mitochondria fragmentation (Fig. 3c), and impairs cyt-C release from mitochondria (Fig. 3d). Mitochondria fragmentation and cyt-C release are also impaired in primary hPBT cells silenced for either ERK1/2 or JNK1 (Fig. S3A–F). This confirms the key role of these kinases in controlling mitochondria alterations during AICD progression.

Since an extensive crosstalk between these kinases has been reported in different contexts [24], we decided to look at whether they modulate each other during AICD. JNK1 silencing does not affect ERK1/2 activation during AICD (Fig. 3e and S3G), and ERK1/2 silencing does not modulate JNK1 activation immediately after TCR engagement (Fig. S3H). However, we found that ERK1/2 is required to sustain JNK1 activation by phosphorylation at later AICD time points, when mitochondria structural alterations begin (Fig. 3f).

Next, we investigated how these kinases modulate Drp1 and Bim activity during AICD. Interestingly, both proteins are able to phosphorylate Drp1 on Ser616. Indeed, double silencing of both JNK1 and ERK1/2 reduces Drp1 phosphorylation more than single silencing in Jurkat cells (Fig. 3g). Furthermore, although ERK1/2 sustains JNK1 activity, Drp1 phosphorylation on Ser616 is not restored when active MKK7B2-JNK1a1 is expressed in siERK1/2-silenced Jurkat cells (Fig. 3h), suggesting that ERK1/2 directly phosphorylate Drp1 independently from JNK1 regulation. Last, we confirmed that silencing of either JNK1 or ERK1/2 reduces Drp1 phosphorylation also upon AICD induction (Fig. 3i). Regarding Bim, we found that only ERK1/2, and not JNK1, is required for Bim-L and -S upregulation during AICD (Fig. 3j).

These data suggest that, following TCR engagement, ERK1/2 and JNK1 are required to initiate the mitochondria structural alterations responsible for the cell death induction. Specifically, ERK1/2 upregulates the expression of shorter Bim-L and -S isoforms and sustains JNK1 activity. Together, JNK1 and ERK1/2 cooperate to mediate Drp1 phosphorylation on Ser616.

### Drp1 and Bim mediate cytochrome-C release downstream of MAPK pathway activation

**Fig. 4:**
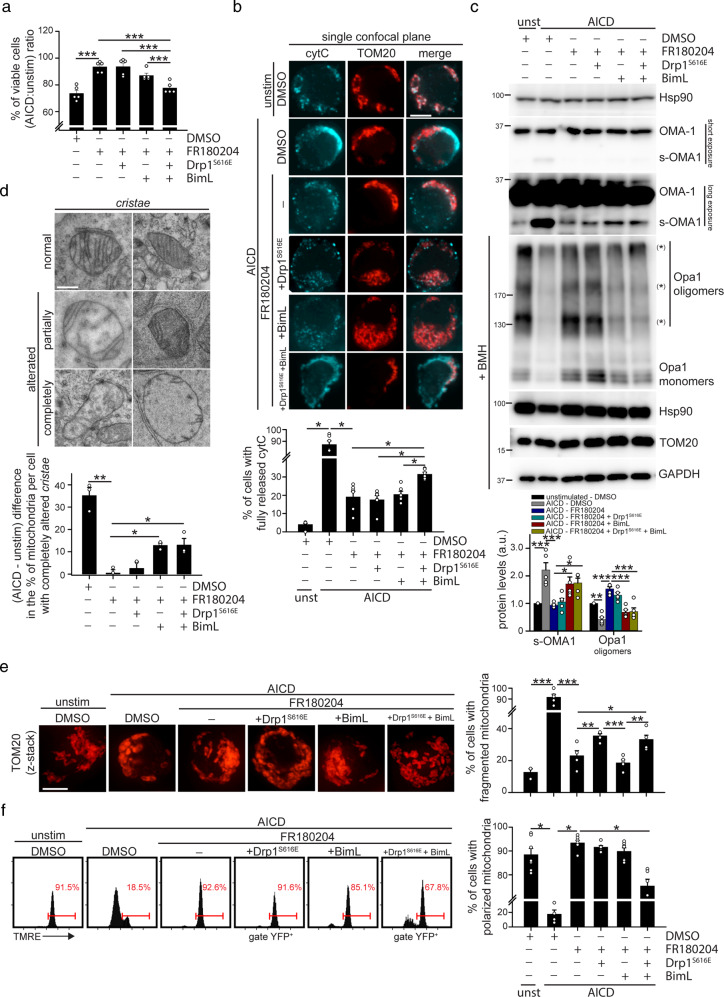
Drp1 and Bim mediate AICD progression downstream of JNK1 and ERK1/2. Jurkat cells have been transfected with the indicated plasmids (pEYFP-Drp1-S616E and Flag-Bim-L). 24 h after transfection, Jurkat cells have been stimulated for AICD in presence or not of FR180204, as indicated. **a** Relative viability (AICD:unstimulated ratio) of YFP-Drp1 and Bim-L transfected Jurkat cells after 32 h from AICD induction (percentage of annexinV^neg^7AAD^neg^ cells assessed by flow cytometry). Cells have been gated on YFP-positive cells when transfected with YFP-Drp1-expressing plasmid (*n* = 5). **b** Representative single z-stack images of mitochondria morphology (TOM20) and cytochrome-C (light blue) 28 h after AICD induction in Jurkat cells transfected with YFP-Drp1 and Bim-L. Quantification of the percentage of cells with fully released cyt-C is reported in the graph below (*n* = 6). **c** Expression levels of the indicated (phospho)-proteins 28 h after AICD induction in Jurkat cells transfected with YFP-Drp1 and Bim-L. Opa1, Hsp90, TOM20 and GAPDH have been evaluated in samples treated with BMH (+BMH) to isolate Opa1 oligomers, which are indicated with an asterisk (*). Quantifications of the amount of s-OMA1 and Opa1 oligomers are reported in the graph below (*n* = 5). One-way ANOVA have been performed to analyze s-OMA1 levels. One-way ANOVA on repeated measurements have been performed to analyze Opa-1 oligomers. **d** Representative electron micrographs of Jurkat cells transfected with YFP-Drp1 and Bim-L 28 h after AICD induction. Mitochondria have been categorized according to *cristae* alterations (normal, partially or completely altered) and the quantification of the (AICD - unstimulated) difference in the percentage of mitochondria belonging to the completely altered-*cristae* category is reported in the graph below (*n* = 3). **e**. Representative images of mitochondria morphology (TOM20, confocal z-stack reconstructions) 28 h after AICD induction in Jurkat cells transfected with YFP-Drp1 and Bim-L. Quantification of the percentage of cells with fragmented mitochondria in each condition is reported in the graph on the right (unstim: *n* = 3; AICD: *n* = 5). **f** TMRE profile by flow cytometry in Jurkat cells transfected with YFP-Drp1 and Bim-L 28 h after AICD induction in presence or not of FR180204. Percentage of cells with polarized mitochondria are quantified in the graph on the right (*n* = 7). Cells have been gated on YFP-positive cells when transfected with YFP-Drp1-expressing plasmid. Data are shown as mean ± SEM. Scale bar, 10 μm in **b**, **e**, 0.4 μm in **d**. Significance is indicated as follows: *p < 0.05; **p < 0.01; ***p < 0.001.

We next asked if Drp1 and Bim activation downstream of JNK1 and ERK1/2 was sufficient to induce cell death upon AICD induction. Interestingly, we observed that neither single active Drp1-S616E, nor single Bim-L overexpression alone restores cell death in AICD-induced Jurkat cells in presence of FR180204 inhibitor (Fig. S4A–D). However, their double transfection (Fig. S4E) efficiently restores cell death (Fig. 4a and S4F), cleavage of PARP (Fig. S4G), and cyt-C release (Fig. 4b and S4H) in AICD-induced FR180204-treated Jurkat cells, this indicating that ERK1/2-JNK1 pathway mediates AICD induction through Drp1 and Bim modulation.

Furthermore, we asked which events related to cyt-C release are specifically induced by Drp1 and/or Bim activation, upon AICD. To this aim, we assessed the two main events known to be involved in cyt-C release: the disassembly of mitochondrial *cristae*, which allows cyt-C translocation toward the mitochondria intermembrane space, and the Mitochondrial Outer Membrane Permeabilization (MOMP), which allows cyt-C to cross the OMM and to accumulate in the cytosol [25]. Interestingly, Drp1 and Bim can regulate either one or both steps in different cell types during apoptosis [14, 21, 26, 27]. Here, we found that *cristae* disassembly is mainly regulated by Bim, acting through the modulation of Opa1 oligomerization. Opa-1 protease OMA1, but not YME1L one (Fig. S4I), is activated, and Opa-1 oligomers are cleaved, during AICD induction (Fig. 4c), with both these events depending on Bim activity. Indeed, consistent with a previous report in a different cell system [26], we found that Bim-L over-expression restores the accumulation of the short and active OMA1 form, and the degradation of Opa-1 oligomers, in AICD-induced FR180204-treated Jurkat cells (Fig. 4c). This is true either with the single overexpression of only Bim-L or in combination with the active Drp1-S616E construct, which, on the contrary, is not able to restore these events alone. Consistently, Bim-L overexpression (either alone or in presence of active Drp1-S616E), but not Drp1-S616E alone, restores the complete disappearance of mitochondrial *cristae* upon AICD induction in FR180204-treated Jurkat cells (Fig. 4d). These data indicate that Bim, and not Drp1, is mainly responsible for the massive disappearance of *cristae* during AICD. In line with this, we previously showed that Drp1 is mainly involved on swelling the *cristae*-junctions more than on regulating the *cristae* number during AICD [12].

On the contrary, active Drp1-S616E restores the fragmentation of the mitochondrial network in AICD-induced and FR180204-treated Jurkat cells (Fig. 4e), which is a prerequisite for MOMP [14]. The latter normally requires Bax/Bak oligomerization on the OMM, in which Bim plays a positive role, too [21]. Consistently, MOMP is restored in AICD-induced and FR180204-treated Jurkat cells only when both Drp1-S616E and Bim-L are over-expressed, as assessed by looking at the depolarization of mitochondrial membrane potential (MMP) (Fig. 4f), as readout of MOMP. Last, as previously reported [28], Bax is not detectable in Jurkat cells, regardless of AICD (Fig. S4J). By contrast, Bak oligomerizes upon AICD (Fig. S4K), and its silencing by specific siRNAs (Fig. S4L) significantly reduces cell death in these conditions (Fig. S4M), thereby confirming its role during AICD progression.

In sum, ERK1/2 and JNK1 activate Drp1 and Bim to drive mitochondria alterations leading to cell death upon AICD. Specifically, Bim is responsible for the disassembly of the mitochondrial *cristae* through OMA1-mediated cleavage of Opa-1 oligomers. In addition, Bim cooperates with Drp1 to mediate the MOMP to fully release cyt-C in the cytosol.

### Downregulation of MAPK-dependent protein Bim may correlate with defective AICD response in T-ALL cells

**Fig. 5:**
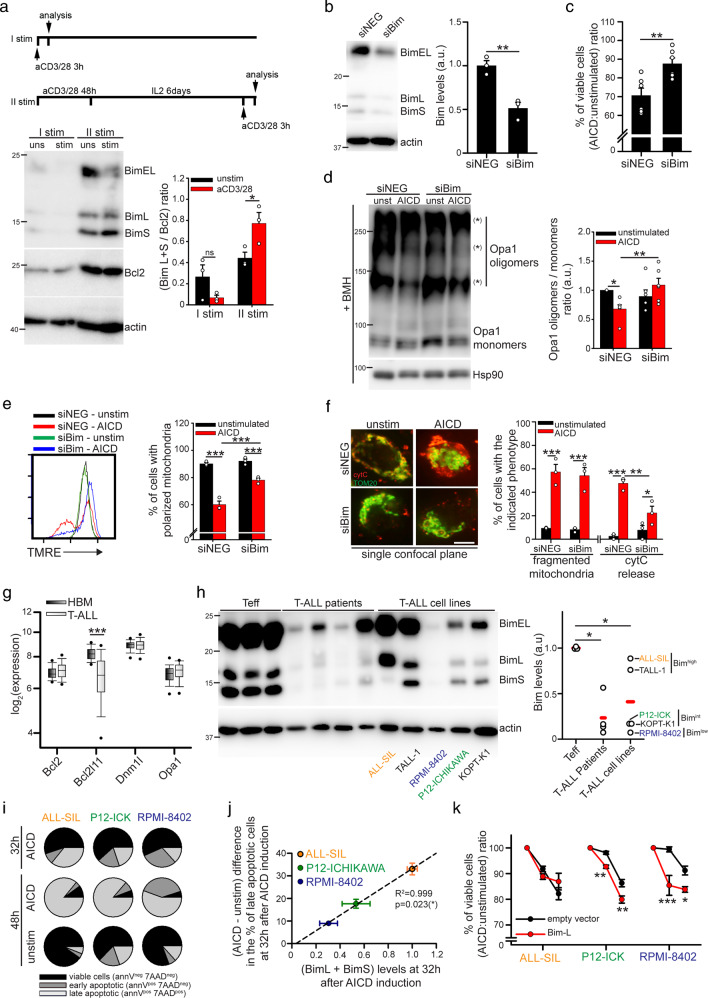
Bim is required for AICD progression in normal and T-ALL cells. **a** Expression levels of the indicated proteins in naïve hPBT stimulated once, for 3 h (I stim), or twice (II stim) with anti-CD3 (plate-coated) and anti-CD28 antibodies. For the second stimulation, cells have been pre-activated with anti-CD3 (plate-coated) and anti-CD28 antibodies for 48 h and expanded for 6 days in IL2-containing medium. The quantification of the ratio between Bim-L/S protein isoforms and Bcl2 protein is reported for each condition in the graph on the right (*n* = 3). **b–f** AICD has been induced in Jurkat cells silenced for Bim (checked by western blot in B, *n* = 3). Relative viability (AICD:unstimulated ratio) at 30 h upon AICD induction is indicated in C (*n* = 6), western blot analysis of the Opa1 oligomers at 30 h in **d** (*n* = 6; relative quantification on the right), TMRE staining at 26 h by flow cytometry in **e** (*n* = 3; relative quantification on the right), and immunofluorescence analysis of mitochondria morphology (TOM20) and cyt-C localization at 30 h, in single confocal plane, in **f** (*n* = 3; relative quantifications of percentage of cells with fragmented mitochondria, or released cyt-C, on the right). **g** Expression levels of the indicated genes from T-ALL and healthy bone marrows (HBM) obtained from Leukemia MILE Dataset and analyzed through BloodSpot (HBM: *n* = 73; T-ALL: *n* = 174). **h** Western blot analysis of Bim levels in healthy human Teff cells (stimulated once, and expanded 6 days with IL2), T-ALL patients and indicated T-ALL cell lines. Relative quantification of total Bim levels is reported in the graph on the right (*n* = 3 controls; 4 patients; 5 cell lines ALL-SIL; TALL-1; RPMI-8402; P12-ICHIKAWA (P12-ICK); KOPT-K1). **i** Pie plots showing the mean percentage of viable (annV^neg^7AAD^neg^; in black), early apoptotic (annV^pos^7AAD^neg^; in dark gray) and late apoptotic cells (annV^pos^7AAD^pos^; in light gray) at the indicated time points after AICD induction in ALL-SIL, P12-ICHIKAWA (P12-ICK) and RPMI-8402 cells from three independent experiments (*n* = 3). *P* values for the differences among the three cell lines at 32 h and 48 h after AICD induction are here indicated (*n* = 3). At 32 h: early apoptotic SIL vs RPMI *p* < 0.001 (***), SIL vs P12 *p* < 0.001 (***), P12 vs RPMI *p* < 0.001 (***); late apoptotic SIL vs RPMI *p* < 0.001 (***), SIL vs P12 *p* = 0.002 (**), P12 vs RPMI *p* = 0.037 (*). At 48 h: early apoptotic SIL vs RPMI *p* < 0.001 (***), SIL vs P12 *p* = 0.049 (*), P12 vs RPMI *p* < 0.001 (***); late apoptotic SIL vs RPMI *p* < 0.001 (***), SIL vs P12 *p* = 0.67, P12 vs RPMI *p* = 0.037 (*). **j** Linear regression between Bim-L + Bim-S protein levels (*n* = 11) and (AICD - unstimulated) difference in the percentage of late apoptotic (annV^pos^7AAD^pos^) cells 32 h after AICD induction in ALL-SIL, P12-ICHIKAWA and RPMI-8402 cells (*n* = 3). Representative western blot of Bim protein levels is reported in Fig. S5B. **k** Relative viability (AICD:unstimulated ratio) at the indicated time points after AICD induction in ALL-SIL, P12-ICHIKAWA (P12-ICK) and RPMI-8402 cells transfected either with pCDNA3 empty vector or with pCDNA3-Flag-Bim-L plasmid (SIL and P12: *n* = 3; RPMI: *n* = 6). Data are shown as mean ± SEM. Scale bar, 10 μm in **f**. Significance is indicated as follows: *p < 0.05; **p < 0.01; ***p < 0.001; ns not significant.

JNK1, ERK1/2 and Drp1 are activated both during the first proliferation-driving and the second AICD-inducing TCR-dependent stimulation of T cells [8, 29, 30]. By contrast, Bim has mainly a pro-apoptotic role [31]. Indeed, Bim levels are specifically upregulated in hPBT cells only during the second AICD-triggering TCR stimulation (Fig. 5a), which is decisive as a killing signal in an ERK-dependent way (Fig. 3j), and not during the first TCR stimulation, that in absence of a second, close, stimulation is a mere pro-survival proliferative signal (Fig. 5a). Therefore, the upregulation of Bim shorter isoforms seems to be a key event for T cells to discern between proliferation and death. Indeed, by preventing Bim upregulation upon TCR stimulation through siRNA-mediated silencing in Jurkat cells (Fig. 5b), we are able to inhibit the induction of cell death (Fig. 5c). As expected, this is associated with an impaired disassembly of Opa1 oligomers (Fig. 5d), a defective MMP depolarization (Fig. 5e), and a reduced cyt-C release (Fig. 5f), without affecting mitochondria fragmentation (Fig. 5f).

Given the key role of Bim in regulating the AICD response, we wondered how its expression levels were modulated in T-cell Acute Lymphoblastic Leukemia (T-ALL) cells, which are frequently characterized by a reduced response to AICD induction, this contributing to their acquisition of a cancerous state. Interestingly, a survey on the Leukemia MILE Dataset shows that T-ALL primary cells express lower mRNA levels of *Bcl2l11* (Bim) compared with healthy bone marrow control tissue while *Dnm1l* (Drp1), *Opa1* and *Bcl2* levels do not vary (Fig. 5g). Consistently, T-ALL cells from pediatric patients express lower Bim protein levels compared with healthy effector T cells (Fig. 5h), a trend confirmed also by RRPA analysis (Fig. S5A). Among T-ALL cell lines, Bim is nearly normal expressed in ALL-SIL and TALL-1 cells, partially downregulated in P12-ICHIKAWA and KOPT-K1 cells, and almost absent in RPMI-8402 cells (Fig. 5h) compared with healthy human effector T cells. Therefore, T-ALL cells are frequently characterized by lower Bim levels, when compared with healthy T cells.

Next, we asked whether the resistance of T-ALL cells to AICD might be partly due to such Bim downregulation. To test this hypothesis, we induced AICD in Bim^high^ ALL-SIL, Bim^int^ P12-ICHIKAWA and Bim^low^ RPMI-8402 cells. Among these three cell lines, Bim^high^ ALL-SIL cells die faster, followed by Bim^int^ P12-ICHIKAWA cells, while Bim^low^ RPMI-8402 cells show the slowest death response upon AICD induction (Fig. 5i). These data revel a strong positive correlation between Bim-L/S levels and cell death response 32 h after AICD induction (Fig. 5j and S5B). To further investigate whether such reduction in AICD response in some T-ALL cell lines could be accounted for their lower Bim expression, we induced AICD after overexpressing Bim-L (Fig. S5C). Interestingly, Bim-L overexpression does not increase cell death rate upon AICD induction in ALL-SIL cells and only slightly in P12-ICHIKAWA ones, with respectively prominent and intermediate original levels of Bim-L/S (Fig. 5k). On the contrary, we found that cell death rate is greatly increased by Bim-L overexpression in RPMI-8402 cells, whose endogenous Bim levels are very low (Fig. 5k).

These data indicate that upon TCR stimulation the upregulation of Bim shorter isoforms is a key event triggering AICD induction. Furthermore, the lower expression of Bim in T-ALL cells may contribute to their resistance to AICD induction.

### Drugs targeting JNK and ERK promotes T cell accumulation inside a solid tumor mass

**Fig. 6:**
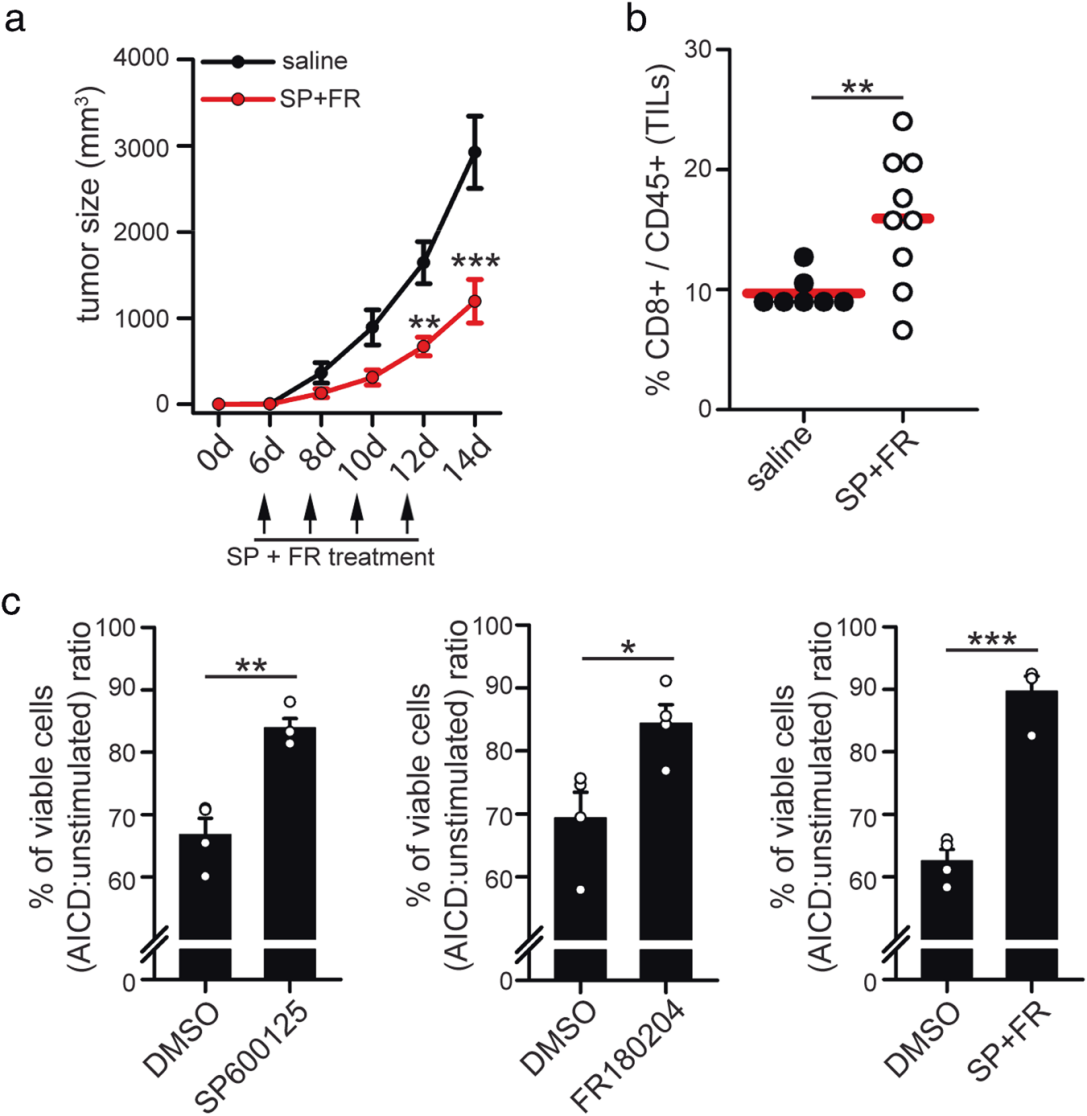
Drugs targeting MAPK proteins reduces tumor growth in vivo and increases T cell survival. **a, b** 5*10^5^ MC38 tumor cells have been inoculated s.c. into the right flank of WT mice. On the indicated days, mice have been injected i.p with SP600125 and FR18024, and tumor volume assessed (**a**, *n* = 7 saline; *n* = 9 SP + FR-treated). After 14 days, TILs have been isolated from tumor mass and the percentage of CD8^+^ T cells among all CD45^+^ TILs quantified by flow cytometry (**b**, *n* = 7 saline; *n* = 9 SP + FR-treated). **c** T cells have been isolated from the spleen of 14 days-old MC38-derived tumor-bearing WT mice and cultured in vitro for 10 days in presence of UV-irradiated MC38 cells, plus IL2 and IL15, to expand tumor-reactive T cells. After 10 days, CD8^+^ T cells have been magnetically purified and re-stimulated for the indicated time with plate-coated anti-CD3 antibodies to induce AICD in presence or not of SP600125 and/or FR180204. Graphs show the relative viabilities (AICD:unstimulated ratio) of murine T cells stimulated 6 h for AICD (percentage of annexinV^neg^7AAD^neg^ cells assessed by flow cytometry) (*n* = 4). Data are shown as mean ± SEM. Significance is indicated as follows: *p < 0.05; ***p < 0.001.

MEK inhibitors effectively reduce tumor growth in mice, partly by inhibiting tumor-infiltrating CD8^+^ T cell apoptosis [7]. Since MEK proteins regulate JNK and ERK kinases, key AICD regulators, we asked whether treatment with JNK and ERK inhibitors could show a similar effect. WT mice were inoculated s.c. with MC38 tumor cells (murine adenocarcinoma) and then i.p. injected with SP600125 and FR180204. Interestingly, such treatment effectively reduces tumor growth (Fig. 6a) and increases the percentage of CD8^+^ T cells among all CD45^+^ leukocytes recovered from the tumor mass (Fig. 6b). Furthermore, we observed that SP600125 treatment inhibits cell death (Fig. S6A), mitochondria fragmentation and cyt-C release (Fig. S6B) in freshly isolated and in vitro activated murine T cells. Also, in the presence of SP600125 and FR180204, either alone or in combination, AICD induction is prevented in T cells isolated from spleen of MC38-derived tumor-bearing mice and chronically exposed in vitro to MC38 tumor cells to select cells responding to tumor antigens (Fig. 6c).

These data indicate that pharmacological inhibition of ERK and JNK kinases effectively inhibits tumor growth in mice, and prevents apoptosis of chronically stimulated T cells.

## Discussion

**Fig. 7:**
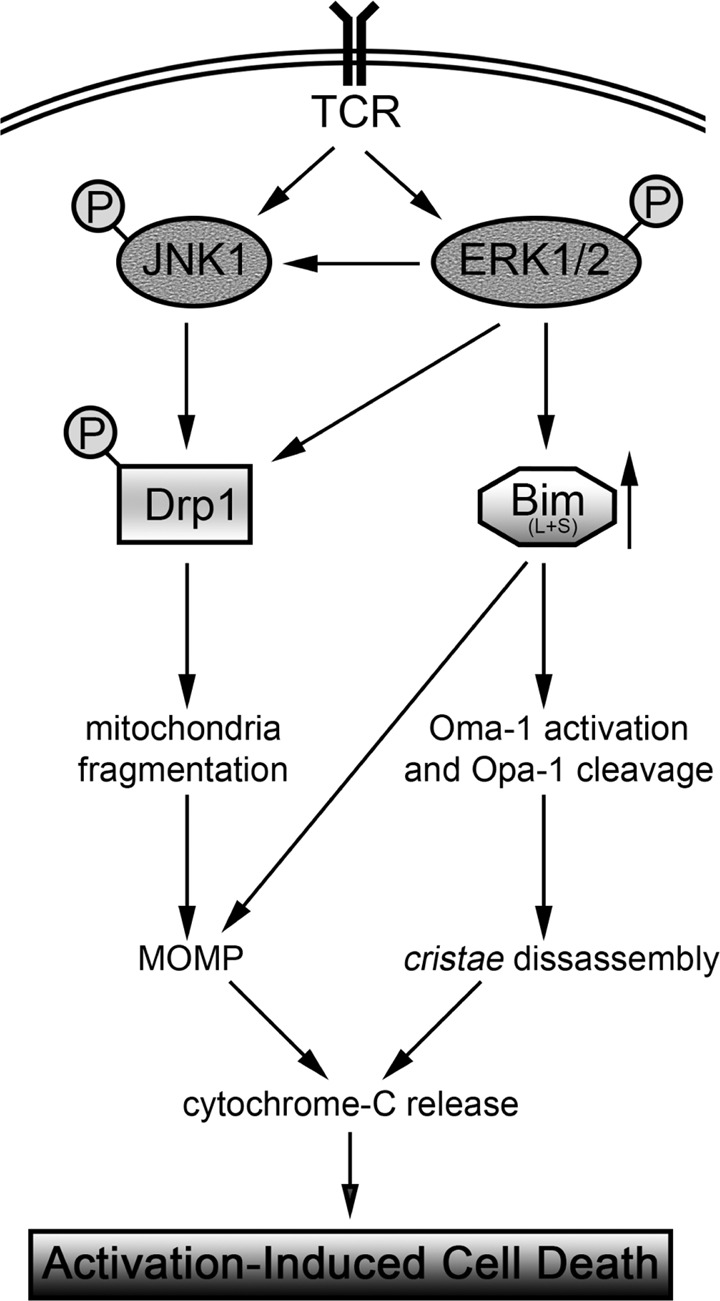
Schematic representation of the molecular pathway leading to cell death on AICD. Diagram illustrating the molecular events occurring after TCR engagement and leading to AICD induction. Upon TCR triggering, both JNK1 and ERK1/2 are phosphorylated and activated. ERK1/2 sustains JNK1 activation and promotes the upregulation of Bim isoforms L and S. In turn, Bim activates the protease OMA1, which cleaves Opa-1 oligomers, promoting *cristae* disassembly. Together, JNK1 and ERK1/2 phosphorylate Drp1 on Ser616, promoting its activation. Active Drp1 fragments mitochondria, which is a prerequisite to mediate the Mitochondrial Outer Membrane Permeabilization (MOMP) in combination with Bim. *Cristae* disassembly and MOMP promote cytochrome-C release in the cytosol, leading to cell death by activating caspases.

Our findings indicate that the extrinsic FasL/Fas pathway is not involved in the crucial early mitochondria alterations upon AICD induction in T cells but is required only in a later phase probably to amplify the cell death cascade. On the contrary, we found that MAPK proteins JNK1 and ERK1/2 are key pro-apoptotic factors, regulating the mitochondrial structural alterations typically orchestrating AICD in T cells (Fig. 7). Furthermore, these two proteins promote the release of cyt-C from mitochondria independently from (and before) the activation of the extrinsic FasL/Fas pathway. ERK1/2 upregulate the pro-apoptotic Bim-L and Bim-S isoforms and cooperate with JNK1 to activate Drp1. In turn, Bim and Drp1 execute the cell death cascade. Specifically, Bim activates OMA1, which mediates cleavage of Opa-1 oligomers and consequently *cristae* disassembly. Presumably, Bim-dependent OMA1 activation occurs through Bak [26], which act independently from its role during MOMP [32]. Then, Drp1 and Bim mediate the MOMP, allowing full cyt-C release in the cytosol, where it activates caspases.

MAPK proteins regulate Bim activity in several ways. JNK-dependent Bim phosphorylation on Thr56 promotes Bim translocation from microtubules towards mitochondria [18]. However, this site is already phosphorylated in T cells [33] and is not modulated during AICD. Furthermore, ERK and JNK phosphorylate Bim-EL on Ser69, so promoting its proteasome-mediated degradation [34, 35], as we also observed during AICD induction. Interestingly, shorter Bim-L and Bim-S isoforms lack the degradation-related Ser69 and accumulate during AICD. Although both JNK and ERK may modulate Bim transcription in different contexts, we found that ERK is mainly responsible for Bim upregulation upon AICD in T cells, similarly to what occurs in cerebral endothelial cells [36]. Instead, in neuronal and hepatic cells, Bim upregulation is mainly controlled through a modulation of JNK levels [37, 38].

ERK and JNK proteins cross-regulate each other in multiple ways in different contexts. JNK can either inhibit [24] or sustain [39] ERK activation in different settings. Here, we observed that ERK1/2 sustains JNK1 activation in T cells upon AICD induction, and both cooperate positively to mediate cell death. Although additional MAPK proteins have been involved in promoting T-cell death, they act by other mechanisms. ERK5 controls the transcription of the proapoptotic factor Nur77 [10]. p38 sustains Bcl-2 expression in CD8^+^ T cells and it is required for their survival [40]. On the contrary, JNK2 frequently shows an anti-apoptotic effect [41] and indeed does not modulate mature T-cell death [42].

In addition, there is a big debate about the exact timing of *cristae* alterations during apoptosis in relation to caspases activation, especially FasL/Fas pathway-dependent caspase-8. Interestingly, in the context of a physiological apoptosis, i.e., AICD, the disruption of Opa-1 oligomers drives *cristae* disassembly before caspases activation, whose modulation does not prevent such alterations. Therefore, at least in T lymphocytes, *cristae*-remodeling is caspase-independent and instead precedes caspase activity. Whether this *timing* is true only in T cells or could be extended to other cell systems during apoptosis needs further work to be firmly established.

Physiologically, generation of memory T cells rely on efficient clearance of effector T cells via AICD and the survival of a small population of T cells, which will form the long-lived memory T cell pool able to sustain stronger and quicker response if challenged by the original antigen. Maintenance of tight mitochondrial *cristae* organization plays a pivotal role in this process [43]. Interestingly, an original report from a Bim KO mouse model showed a Bim-dependent resistance to certain type of apoptosis induction in T cells and development of autoimmunity [44]. Here, we provide a hint of how Bim deficiency may result in autoimmunity via T cells escaping AICD.

Last, to acquire a cancerous state, leukemia T cells need evading the homeostatic mechanisms set up by the organism to avoid undesired hyper-proliferation of cells. Since AICD is responsible to keep under control the unwanted proliferation of chronically active T cells, leukemia T cells need to acquire the ability to evade AICD-mediated control. We found here that ERK1/2, JNK1, Drp1, and Bim constitute a core pathway executing AICD. Interestingly, we also found that primary T-ALL cells show a downregulation of one of these proteins, Bim, with this conferring them resistance to AICD. Although we cannot exclude that alterations in other Bcl2 family proteins may modulate in more complex ways the T-ALL response to multiple apoptotic stimuli, Bim downregulation may represent one of the causes conferring transformed T cells a cancerous phenotype.

In sum, we found that a MAPK-mediated pathway, and not the FasL/Fas-pathway, mainly controls early mitochondria morphological alterations upon AICD induction in T cells, by regulating both Drp1 and Bim activity, and leading to cell death. Furthermore, Bim expression is altered in T-ALL cells, most likely contributing to their cancerous characteristics.

## Materials and methods

### Human samples

Peripheral blood samples were purified from buffy coats of healthy adult volunteer blood donors (independently of sex and age) under procedures approved by Institutional Review Board of Bambino Gesù Children’ Hospital (Approval of Ethical Committee N° 969/2015 prot. N° 669LB), including informed consensus for research purpose. Blood cells were incubated with RosetteSep Human T cell enrichment cocktail antibody mix (StemCell 15061). Unlabeled Human Peripheral Blood T (hPBT) cells were isolated by density gradient over Lymphoprep (StemCell 07811), with centrifugation for 20 min at 1200rcf. Then T cells have been collected, washed and used for subsequent analyses. Human leukemia cell lines ALL-SIL, P12-ICHIKAWA, RPMI-8402 and TALL-1 were purchased from DSMZ (Braunschweig, Germany). Protein lysates were obtained from primary samples of patients affected by T-ALL at diagnosis previously used and published by Serafin et al. [45]. Caspase-8 KO Jurkat cells are a gift from Dr Fulda Simone (Klinikums der Goethe-Universität Frankfurt) and Dr John Blenis (Dept. Cell Biology, Harvard Medical School). Fas insensitive Jurkat cells are a gift from Dr Olivier Micheau (INSERM) [46]. KOPT-K1 cells are a kind gift from Dr S. Indraccolo (Istituto Oncologico Veneto IRCCS, Padua, Italy). Jurkat cells were an in-house stock. All cell lines were routinely tested for mycoplasma contamination.

### Mice

C57BL/6 WT mice were bred and maintained under conventional conditions at the Plaisant Srl (Castel Romano) and IRCCS Fondazione Santa Lucia Animal Facilities. They were kept in cages of no more than 5–6 mice each, divided by sex, under 12 h/12 h light/dark cycle, with standard temperature, humidity and pressure conditions according to FELASA guidelines. Small red squared mice house and paper were used for cage enrichment. From breeding until weaning a specific food (richer in fat and protein than the standard one used for maintenance) were used in all cages. Mice health was monitored daily by veterinary staff and health analysis for pathogens were performed every three months according to FELASA guidelines. Weaning was performed not earlier than 28 post-natal days. All mice were sacrificed by neck dislocation at 2–3 months of age. All efforts were made to minimize animal suffering and to reduce the number of mice used, in accordance with the European Communities Council Directive of 24 November 1986 (86/609/EEC). The mice protocol has been approved by the IRCCS Fondazione Santa Lucia and the Plaisant Srl Ethical Committees as well as by the Italian Ministry of Health (Authorizations #459/2015-PR and #186/2020-PR). It has been written following the ARRIVE Guidelines, and the numeric details have been chosen following the criteria described in The National Centre for the Replacement, Refinement and Reduction of Animals in Research (NC3Rs). Sample size for the experiments performed has been established using power analysis method. Experiments involving growth of tumor cells in mice where performed using male mice.

### Cell cultures and reagents

Murine T cells have been isolated from spleen using 70 mm Cell Strainers (Corning 431751) and cultured in RPMI 1640 medium (Thermo Fisher 21875) supplemented with 10% Fetal Bovine Serum (Thermo Fisher 10270), 2 mM L-glutamine (Thermo Fisher 25030081), 100 U/ml penicillin/streptomycin (Thermo Fisher 15140130), 1x GIBCO MEM Non-essential amino-acids (Thermo Fisher 11140035), 1 mM Sodium pyruvate (Thermo Fisher 11360039), 100 mg/ml Gentamycin (Thermo Fisher 15750045), and 50 µM β-mercaptoethanol (Thermo Fisher 31350-010).

MC38 tumor cells (gift from Dr S. Piconese, University of Rome La Sapienza, Rome, Italy) have been cultured in complete DMEM medium (Thermo Fisher 41966052) supplemented with 10% Fetal Bovine Serum (Thermo Fisher 10270), 2 mM L-glutamine (Thermo Fisher 25030081), 100 U/ml penicillin/streptomycin (Thermo Fisher 15140130), 1x GIBCO MEM Non-essential amino-acids (Thermo Fisher 11140035), 1 mM Sodium pyruvate (Thermo Fisher 11360039), and 50 µM β-mercaptoethanol (Thermo Fisher 31350-010).

HeLa cell line has been cultured in complete DMEM medium (Thermo Fisher 41966052) supplemented with 10% Fetal Bovine Serum (Thermo Fisher 10270), and 100 U/ml penicillin/streptomycin solution (Thermo Fisher 15140130).

WT, Caspase8 KO and FAS-insensitive Jurkat cells have been cultured in RPMI 1640 medium (Thermo Fisher 21875) supplemented with 10% Fetal Bovine Serum (Thermo Fisher 10270), 2 mM L-glutamine (Thermo Fisher 25030081), 100 U/ml penicillin/streptomycin (Thermo Fisher 15140130), 1x GIBCO MEM Non-essential amino-acids (Thermo Fisher 11140035), and 1 mM Sodium pyruvate (Thermo Fisher 11360039).

T-ALL human cell lines ALL-SIL, P12-ICHIKAWA, TALL-1 and RMPI-8402 have been cultured in RPMI 1640 medium (Thermo Fisher 21875) supplemented with 20% Fetal Bovine Serum (Thermo Fisher 10270), 2 mM L-glutamine (Thermo Fisher 25030081), 100 U/ml penicillin/streptomycin (Thermo Fisher 15140130), 1x GIBCO MEM Non-essential amino-acids (Thermo Fisher 11140035), and 1 mM Sodium pyruvate (Thermo Fisher 11360039).

Human Peripheral Blood T (hPBT) cells have been cultured in RPMI 1640 medium (Thermo Fisher 21875) supplemented with 10% Fetal Bovine Serum (Thermo Fisher 10270), 2 mM L-glutamine (Thermo Fisher 25030081), 100 U/ml penicillin/streptomycin (Thermo Fisher 15140130), 1x GIBCO MEM Non-essential amino-acids (Thermo Fisher 11140035), 1 mM Sodium pyruvate (Thermo Fisher 11360039), and 100 mg/ml Gentamycin (Thermo Fisher 15750045).

To induce AICD, Jurkat, ALL-SIL, P12-ICHIKAWA and RMPI-8402 cells have been resuspended in RPMI medium with 1% FBS (instead of 10%) at the final concentration of 600.000 (Jurkat) or 700.000 (SIL, P12, RPMI) cells in 200 µl medium, in 96well plate. Anti-human CD3 antibody (clone OKT3, eBioscience 16-0037-85) has been added at a final concentration of 1 µg/ml and cells have been kept for 30 min on ice. Then, secondary biotin anti-mouse antibody (Sigma B7401) has been added at final dilution of 1:50 and cells kept in the incubator at 37 °C and 5% CO_2_. After 8 h, 100 µl of medium has been removed and replaced with RPMI complete medium (with 10% FBS).

To induce AICD in hPBT cells, freshly isolated cells have been activated o.n. with 1 mg/ml PHA (Millipore M5030) and then expanded for 6 days in vitro with 12.5 ng/ml IL2 (eBioscience 14-8028-81). Then cells have been resuspended in RPMI medium with 1% FBS (instead of 10%) at the final concentration of 500.000 cells in 200 µl medium, in 96well plates previously coated o.n. with anti-human CD3 antibody (clone OKT3, eBioscience 16-0037-85), at the final concentration of 10 µg/ml in Dulbecco PBS with Ca^2+^ Mg^2+^ (Thermo Fisher 14040133). Cells have been immediately kept in the incubator at 37 °C and 5% CO_2_. AICD has been similarly induced in murine T cells, with the only difference of using plate-coated anti-mouse CD3 antibody (clone 145-2C11, eBioscience 14-0031-86). When indicated, the following drugs have been added during AICD induction for the whole period of the assay: 30 µM SP600125 (Tocris 1496), 30 µM FR180204 (Tocris 3706), 1 µM actinomycinD (Tocris 1229), 10 µM MG132 (Sigma M7449), 2 µM z-VAD-FMK (G-Biosciences CPI-00G). Cell death was also induced by treatment with 2 µM staurosporine (Sigma S4400), 50 µM etoposide (E1383-25MG, Sigma) and 20 ng/ml anti-CD95 antibody (CH11, CD95 activating clone, Sigma 05-201) for 4 h.

To evaluate Bim levels between first and second stimulation, naïve hPBT isolated using Naïve Pan T cell Isolation Kit Human (Miltenyi 130-097-095) have been stimulated in vitro with 10 µg/ml anti-CD3 plate-coated antibody (clone OKT3, eBioscience 16-0037-85) and 2 µg/ml anti-CD28 antibody (eBioscience 16-0289-85) for 48 h. Then, cells have been expanded for 6 days in vitro with 12.5 ng/ml IL2 (eBioscience 14-8028-81) and re-stimulated for 3 h as in the first stimulation.

### Cell transfection

Jurkat, RMPI-8402, P12-ICHIKAWA and ALL-SIL cells have been electroporated using Neon Transfection System (Thermo Fisher) following manufacturer instructions and kept o.n. in antibiotics-free medium before being washed and used for further experiments. HeLa cells were transiently transfected with Lipofectamine 2000 (Thermo Fisher 11668030). The following plasmids have been used: pEYFP-C1-Drp1 (Addgene 45160), pEYFP-C1-Drp1-pS616A, pEYFP-C1-Drp1-pS616E (previously described [8]), pCDNA3-Flag-BimL (Addgene 24233), pCDNA3-Flag-MKK7B2JNK1a1 (Addgene 19726), pCDNA3-JNK-DN (kind gift from Jennifer y. Zhang [47]), and pCDNA3-mtYFP (previously described [12]). The following siRNA have been used: siERK1/2 (Cell Signaling 6560), siJNK1 (Santa Cruz sc-29380), siBim (Cell Signaling 6461), siNEG (Santa Cruz sc-37007) and siBak (Cell Signaling 6486).

To generate pEYFP-C1-Drp1-pS616A plasmid from pEYFP-C1-Drp1 one the following primers have been used: Fw 5’-attccaattatgccagccgctccacaaaaaggtcatgccgtgaacctgctagatgtgccag-3’; Rv 5’-acggcatgacctttttgtggagcggctggcataattggaatgggttttgatttttcttctg -3’.

### Western Blot

Western blot were performed as previously described [8]. To isolate Opa1 oligomers, cells have been pre-treated with 1 mM BMH (bismaleimidohexane, Thermo Fisher 22330) for 20 min at 37 °C and then lysed using RIPA buffer as indicated above (BMH also included in the RIPA buffer at final concentration of 1 mM). The following primary antibodies were used: anti-caspase-8 (Cell Signaling 4790), anti-caspase-9 (Cell Signaling 9520), anti-cleaved caspase-3 (Cell Signaling 9661), anti-tubulin (BD Pharmingen 627902), anti-GRP75 (Santa Cruz sc-133137), anti-actin (Cell Signaling 4970), anti-Bcl2 (Cell Signaling 4223), anti-Drp1 (BD Bioscience 611113), anti-pS616-Drp1 (Cell Signaling 4494), anti-pS637-Drp1 (Cell Signaling 6319), anti-Mfn2 (Abcam ab56889), anti-Mfn1 (Santa Crux sc-50330), anti-Opa1 (BD Bioscience 612607), anti-cJun (Cell Signaling 9165), anti-pSer63 cJun (Cell Signaling 9261), anti-pT183/185-SAPK/JNK (Cell Signaling 4671), anti-pT202/204-ERK1/2 (Cell Signaling4370), anti-SAPK/JNK (Cell Signaling 9252), anti-ERK1/2 (Cell Signaling 4695), anti-Bim (Cell Signaling 2933), anti-pSer69-Bim (Cell Signaling 4585), anti-Flag (Sigma F1804), anti-GAPDH (Cell Signaling 2118), anti-Hsp90 (Cell Signaling 4877), anti-OMA1 (Cell Signaling 95473), anti-TOM20 (Santa Cruz, sc-11415), anti-Bid (Cell Signaling 2002), anti-Bak (Cell Signaling 12105), and anti-Bax (556467, BD Pharmingen). All primary antibody incubations were followed by incubation with appropriated secondary HRP-conjugated antibodies (GE Healthcare or Cell Signaling) in 5% milk plus 0.1% Tween20 (Sigma P2287). Detection of protein signals was performed using Amersham ECL Detection Reagent (GE Healthcare RPN2106) on Amersham hyperfilms (GE Healthcare 28906836) or using Clarity Western ECL substrate (Biorad 170-5061) and Amersham Imager 600. Stripping of the membranes for reprobing has been performed using buffer containing Tween-20 1%, SDS 0.1%, and glycine 0.2 M at pH 2.2 (two washes for 10 min).

### Mitochondria-cytosol fractionation

Cells were resuspended in Permeabilization Buffer (140 mM sucrose, 420 mM mannitol, 10 mM Tris, 2 mM EGTA) plus Proteases and Phosphatases Inhibitor Cocktail (Thermo Fisher 78440) and membranes disrupted by mechanical dissociation using 100-hits glass homogenizer (Potter-Elvehjem). Cells were then centrifuged twice for 10 min at 1000rcf to remove cell nuclei and debris. Supernatant was centrifuged at 12000rcf for 15 min to separate the cytosolic fraction (supernatant) and the mitochondria-containing pellet. For western blot analysis, whole cells and cell fractions were lysed in RIPA buffer (see above) plus Proteases and Phosphatases Inhibitor Cocktail (Thermo Fisher 78440). The flowing primary antibodies were used: anti-Drp1-pSer616 (Cell Signaling 4494), anti-TOM20 (Santa Cruz sc-11415), and anti-tubulin (BD Pharmingen 627902), anti-Drp1 (BD Bioscience 611113), anti-ATPb (ab14730, Abcam), anti-prohibitin (PHB, sc-377037 Santa Cruz), anti-tubulin (86298, Cell Signaling) and anti-cytochrome-C (556433, BD Pharmingen).

### Immunofluorescence

For immunofluorescence staining, cells have been fixed in 4% formaldehyde (Carlo Erba Reagents 415661) 15 min, permeabilized in Triton-X 0.3% (Sigma Aldrich X100) 20 min, blocked 1 h at RT with 1% BSA (Sigma Aldrich A-2153) and immunostained with the following primary anti-mouse antibodies o.n. at 4 °C: anti-TOM20 (Santa Cruz sc-11415), anti-cytochorme-C (BD Pharmingen 556432). All primary antibody incubations have been followed by incubation with appropriated fluorochrome- conjugated secondary antibodies 1 h at RT. Images were acquired using a Perkin Elmer Ultraview VoX microscope and Volocity acquisition software. The mitochondrial network has been always evaluated upon 0.4 mm slices z-stack reconstructions. Cytochrome-C co-localization index with mitochondria have been calculated as previously described [48].

### Reverse phase protein arrays (RPPA)

RPPA were performed as previously described [45]. BIM primary antibody (Cell Signaling 2933) was used at 1:100 dilution.

### Transmission electron microscopy

30 h after AICD induction, cells have been collected and centrifuged for 5 min at 300rcf, as described previously [12]. Samples have been post-fixed in 1% OsO4 and 0.05 M K_3_FeCn_6_ in TEM buffer for 1 h at RT, then washed three times in ddH_2_O. Dehydration has been performed with a series of 70-90-100% ethanol solutions and infiltration with a series of Resin Epon/Propylene oxide gradient (1:1, 2:1, 3:1) for 20–40 min. Then samples have been embedded in pure resin for at least 2 h, placed in wells filled with resin and left at 60 °C for at least 24 h. Samples have been analyzed using CM 100 BioTWIN electron Microscope or Tecnai G2 (FEI) transmission electron microscope operating at 100 kV. Images were captured with a Veleta (Olympus Soft Imaging System) digital camera.

In rescue experiments, cells have been categorized according to *cristae* morphology and distinguished in cells with normal *cristae*, partially disappeared *cristae* and completely disappeared *cristae*. The difference in the percentage of mitochondria per cell showing completely disappeared *cristae* between AICD-induced and control cells has been plotted for all the experimental conditions.

### Flow cytometry

T cells viability has been assayed by annexinV-FITC and 7AAD staining (Biolegend 640922) or annexinV-APC and 7AAD staining (Biolegend 640930) according to manufacturer protocol. For evaluation of mitochondrial membrane potential, 1 mM TMRE (Thermo Fisher T669) has been added for 30 min and then, after washing, the cells have been analyzed by flow cytometry. When cells transfected with pEYF-Drp1-(S616E/A) plasmids have been analyzed, cells have been gated on YFP expression. To evaluate caspase activity, cells treated as indicated for AICD induction were incubated 1 h with CaspGLOW Fluorescein Active Caspase-3/8/9 staining kit (Gentaur K189-25) prior to flow cytometry analysis. Acquisitions have been performed using BD Accuri C6 and BD FACSCelesta cytometers. Cleaved PARP has been measured using PE-anti-clPARP antibody (51-9007682, BD Pharmingen) and Foxp3 Transcription Factor Staining Buffer Set (00-5523-00, eBioscience).

### Co-Immunoprecipitation and in vitro kinase assay

HeLa cells were transiently transfected with a vector encoding Flag-MKK7B2JNKa1. After 24 h, equal amounts of proteins were immunoprecipitated using anti-Flag antibody or anti-IgG control. Protein-A agarose beads (Roche, 11719408001) have been added to the samples at 4 °C for 1 h and then, samples were collected by centrifugation after three washes with RIPA buffer. The immunocomplex was evaluated by western blot analysis. For in vitro kinase assay, total extracts from HeLa cells WT (to purify Drp1) or transfected with Flag-MKK7B2JNKa1 (to purify MKK-JNK) were subjected to immunoprecipitation and immunoprecipitates were collected by centrifugation and washed three times with RIPA buffer. Pellets were washed for 5 min with 500 μl Kinase Buffer (20 mM Hepes pH 7.4, 10 mM MgCl2, 25 mM Glycerophosphate, 0.1 Na4VO3, 4 mM NaF, 1 mM DTT). In order to test kinase activity with a non-radioactive method, 1 μl of 10 mM adenosine triphosphate (ATP) and 20 μl of IP-Drp1 substrate were added to the IP-Flag-MKK7B2JNKa1 for 30 min at 30 °C in presence or not of JNK inhibitor 30 µM SP600125 (Tocris 1496). The reaction was stopped by adding NuPage LDS Sample Buffer (Invitrogen NP0008) and denaturizing samples at 95 °C for 5 min.

### Tumor induction

5*10^5^ MCA38 cells were injected subcutaneously into the right flank of two-months old male WT mice. Mice were kept for up to 18 days in animal facility, and tumor growth was monitored twice per week and recorded as (longest diameter)*(shortest diameter)^2^ in cubic millimeters. At days 6, 8, 10 and 12 mice have been inoculated i.p. with 20 mg/kg of SP600125 and FR180204. Mice were randomly subdivided into each experimental group (saline or treated) before drug inoculation (no specific randomization method was used). At day 14, mice were sacrificed and tumors were collected. Tumor tissues were mechanically dissociated over 70 mm-cell strainers, and mononuclear cells were enriched from tumor-derived cell suspensions by 40%/80% Percoll (GE Healthcare GE17-0891-01) density gradient, by collecting cells at the interface between 40 and 80% Percoll solution. Then, mononuclear cells have been analyzed by flow cytometry using anti-mouse-CD8-Alexa488 (Biolegend 100723) and anti-mouse-CD45-PE (Biolegend 103105) antibodies, to measure the percentage of CD8^+^ T cells among all CD45^+^ cells.

Mixed tumor cell lymphocyte culture (MTLC) has been established to expand CD8^+^ T cells from spleen of MC38-bearing mice. Briefly, 5*10^6^ splenocytes have been cultivated in vitro with 20 ng/ml IL2 (R&D System 402-ML-100), 20 ng/ml IL15 (eBioscience 14-8153) and 2.5*10^4^ UV-irradiated MC38 cells for 10 days in 24well plates (last two days without MC38 cells). Then CD8^+^ T cells have been magnetically purified (CD8 + T Cell Isolation kit, mouse, Miltenyi 130-104-075) and re-stimulated for the indicated time with plate-coated anti-CD3 antibodies (clone 145-2C11, eBioscience 14-0031-86) to induce AICD. Cell viability upon AICD has been measured by flow cytometry using annexinV-FITC and 7AAD staining (Biolegend 640922).

### Statistical analysis

In the Figure legends, “*n*” indicates either the number of independent experiments (in vitro cell lines or primary cells) or the number of mice used. Data are expressed as mean ± SEM from at least three independent experiments unless specified otherwise (Microsoft Office Excel and SigmaPlot v12.5 have been used for analysis). All experiments have been performed at least three independent times unless otherwise specified in the figure legends. The number of mice used has been estimated using power analysis method. All the acquisitions of the experiments have been performed blinded without knowing the specific condition of each sample. Comparisons between groups were done using two-tailed Student’s *t* test (two groups) or One-way and Two-way ANOVA (multiple groups, adjustments for pairwise comparisons were performed using Holm-Sidak method). Mann–Whitney Rank Sum Test or ANOVA on ranks have been used if samples did not meet assumptions of normality and/or equal variance. *P* values are indicated in the Figures as follows: *p < 0.05, **p < 0.01, ***p < 0.001.

## Supplementary information

Supplemental Figure 1

Supplemental Figure 2

Supplemental Figure 3

Supplemental Figure 4

Supplemental Figure 5

Supplemental Figure 6

Supplemental Figures Legends

## References

[1] Volpe E, Sambucci M, Battistini L, Borsellino G (2016). Fas-fas ligand: checkpoint of T cell functions in multiple sclerosis. Front Immunol.

[2] Arakaki R, Yamada A, Kudo Y, Hayashi Y, Ishimaru N (2014). Mechanism of activation-induced cell death of T cells and regulation of FasL expression. Crit Rev Immunol.

[3] Zhang J, Xu X, Liu Y (2004). Activation-induced cell death in T cells and autoimmunity. Cell Mol Immunol.

[4] Caruana I, Simula L, Locatelli F, Campello S (2018). T lymphocytes against solid malignancies: winning ways to defeat tumours. Cell Stress.

[5] Ni X, Zhang C, Talpur R, Duvic M (2005). Resistance to activation-induced cell death and bystander cytotoxicity via the fas/fas ligand pathway are implicated in the pathogenesis of cutaneous T cell lymphomas. J Invest Dermatol.

[6] Jäättelä M, Tschopp J (2003). Caspase-independent cell death in T lymphocytes. Nat Immunol.

[7] Ebert PJ, Cheung J, Yang Y, McNamara E, Hong R, Moskalenko M (2016). MAP kinase inhibition promotes T cell and anti-tumor activity in combination with PD-L1 checkpoint blockade. Immunity.

[8] Simula L, Pacella I, Colamatteo A, Procaccini C, Cancila V, Bordi M (2018). Drp1 controls effective T cell immune-surveillance by regulating T cell migration, proliferation, and cMyc-dependent metabolic reprogramming. Cell Rep..

[9] Wang A, Rud J, Olson CM, Anguita J, Osborne BA (2009). Phosphorylation of Nur77 by the MEK-ERK-RSK cascade induces mitochondrial translocation and apoptosis in T cells. J Immunol.

[10] Fujii Y, Matsuda S, Takayama G, Koyasu S (2008). ERK5 is involved in TCR-induced apoptosis through the modification of Nur77. Genes Cells.

[11] Ley R, Ewings KE, Hadfield K, Cook SJ (2005). Regulatory phosphorylation of Bim: sorting out the ERK from the JNK. Cell Death Differ.

[12] Corrado M, Mariotti FR, Trapani L, Taraborrelli L, Nazio F, Cianfanelli V (2016). Macroautophagy inhibition maintains fragmented mitochondria to foster T cell receptor-dependent apoptosis. EMBO J.

[13] Estaquier J, Vallette F, Vayssiere J-L, Mignotte B. The mitochondrial pathways of apoptosis. Adv Exp Med Biol. 2012;942:157–83. 10.1007/978-94-007-2869-1_7 22399422

[14] Simula L, Nazio F, Campello S (2017). The Mitochondrial dynamics in cancer and immune-surveillance. Semin Cancer Biol.

[15] Wan J, Martinvalet D, Ji X, Lois C, Kaech SM, Andrian UHVon (2003). The Bcl-2 family pro-apoptotic molecule, BNIP3 regulates activation-induced cell death of effector cytotoxic T lymphocytes. Immunology.

[16] Guerrero AD, Welschhans RL, Chen M, Wang J (2013). Cleavage of anti-apoptotic Bcl-2 family members after TCR stimulation contributes to the decision between T cell activation and apoptosis. J Immunol.

[17] Zhu L, Yu X, Akatsuka Y, Cooper JA, Anasetti C (1999). Role of mitogen-activated protein kinases in activation-induced apoptosis of T cells. Immunology.

[18] Lei K, Davis RJ (2003). JNK phosphorylation of Bim-related members of the Bcl2 family induces Bax-dependent apoptosis. Proc Natl Acad Sci USA.

[19] Cereghetti GM, Stangherlin A, Martins de Brito O, Chang CR, Blackstone C, Bernardi P (2008). Dephosphorylation by calcineurin regulates translocation of Drp1 to mitochondria. Proc Natl Acad Sci USA.

[20] Hildeman DA, Zhu Y, Mitchell TC, Bouillet P, Strasser A, Kappler J (2002). Activated T cell death in vivo mediated by proapoptotic bcl-2 family member bim. Immunity.

[21] Kim H, Tu H-C, Ren D, Takeuchi O, Jeffers JR, Zambetti GP (2009). Stepwise Activation of BAX and BAK by tBID, BIM, and PUMA Initiates Mitochondrial Apoptosis. Mol Cell.

[22] Yu R, Liu T, Ning C, Tan F, Jin S-B, Lendahl U (2019). The phosphorylation status of Ser-637 in dynamin-related protein 1 (Drp1) does not determine Drp1 recruitment to mitochondria. J Biol Chem.

[23] Miyazawa K (2011). Encountering unpredicted off-target effects of pharmacological inhibitors. J Biochem.

[24] Shen YH, Godlewski J, Zhu J, Sathyanarayana P, Leaner V, Birrer MJ (2003). Cross-talk between JNK/SAPK and ERK/MAPK Pathways. J Biol Chem.

[25] Kalkavan H, Green DR (2018). MOMP, cell suicide as a BCL-2 family business. Cell Death Differ.

[26] Jiang X, Jiang H, Shen Z, Wang X (2014). Activation of mitochondrial protease OMA1 by Bax and Bak promotes cytochrome c release during apoptosis. Proc Natl Acad Sci USA.

[27] Otera H, Miyata N, Kuge O, Mihara K (2016). Drp1-dependent mitochondrial fission via MiD49/51 is essential for apoptotic cristae remodeling. J Cell Biol.

[28] Brimmell M, Mendiola R, Mangion J, Packham G (1998). BAX frameshift mutations in cell lines derived from human haemopoietic malignancies are associated with resistance to apoptosis and microsatellite instability. Oncogene.

[29] D’Souza WN, Chang C-F, Fischer AM, Li M, Hedrick SM (2008). The Erk2 MAPK regulates CD8 T cell proliferation and survival. J Immunol.

[30] Simula L, Campanella M, Campello S (2019). Targeting Drp1 and mitochondrial fission for therapeutic immune modulation. Pharm Res.

[31] Bouillet P, Purton JF, Godfrey DI, Zhang L-C, Coultas L, Puthalakath H (2002). BH3-only Bcl-2 family member Bim is required for apoptosis of autoreactive thymocytes. Nature.

[32] Yamaguchi R, Lartigue L, Perkins G, Scott RT, Dixit A, Kushnareva Y (2008). Opa1-mediated cristae opening is Bax/Bak and BH3 dependent, required for apoptosis, and independent of Bak oligomerization. Mol Cell.

[33] Zhu Y, Swanson BJ, Wang M, Hildeman DA, Schaefer BC, Liu X (2004). Constitutive association of the proapoptotic protein Bim with Bcl-2-related proteins on mitochondria in T cells. Proc Natl Acad Sci.

[34] Luciano F, Jacquel A, Colosetti P, Herrant M, Cagnol S, Pages G (2003). Phosphorylation of Bim-EL by Erk1/2 on serine 69 promotes its degradation via the proteasome pathway and regulates its proapoptotic function. Oncogene.

[35] Leung KT, Li KK-H, Sun SS-M, Chan PKS, Ooi VE-C, Chiu LC-M (2007). Activation of the JNK pathway promotes phosphorylation and degradation of BimEL-a novel mechanism of chemoresistance in T-cell acute lymphoblastic leukemia. Carcinogenesis.

[36] Yin KJ, Lee J-M, Chen SD, Xu J, Hsu CY (2002). Amyloid-beta induces Smac release via AP-1/Bim activation in cerebral endothelial cells. J Neurosci.

[37] Hu K, Huang Q, Liu C, Li Y, Liu Y, Wang H (2019). c-Jun/Bim upregulation in dopaminergic neurons promotes neurodegeneration in the MPTP mouse model of Parkinson’s disease. Neuroscience.

[38] Litwak SA, Pang L, Galic S, Igoillo-Esteve M, Stanley WJ, Turatsinze J-V (2017). JNK activation of BIM promotes hepatic oxidative stress, steatosis, and insulin resistance in obesity. Diabetes.

[39] Kitanaka T, Nakano R, Kitanaka N, Kimura T, Okabayashi K, Narita T (2017). JNK activation is essential for activation of MEK/ERK signaling in IL-1β-induced COX-2 expression in synovial fibroblasts. Sci Rep..

[40] Merritt C, Enslen H, Diehl N, Conze D, Davis RJ, Rincón M (2000). Activation of p38 mitogen-activated protein kinase in vivo selectively induces apoptosis of CD8(+) but not CD4(+) T cells. Mol Cell Biol.

[41] Singh R, Wang Y, Xiang Y, Tanaka KE, Gaarde WA, Czaja MJ (2009). Differential effects of JNK1 and JNK2 inhibition on murine steatohepatitis and insulin resistance. Hepatology.

[42] Sabapathy K, Hu Y, Kallunki T, Schreiber M, David J-P, Jochum W (1999). JNK2 is required for efficient T-cell activation and apoptosis but not for normal lymphocyte development. Curr Biol.

[43] Buck MD, O’Sullivan D, Klein Geltink RI, Curtis JD, Chang CH, Sanin DE (2016). Mitochondrial dynamics controls T cell fate through metabolic programming. Cell.

[44] Bouillet P, Metcalf D, Huang DC, Tarlinton DM, Kay TW, Köntgen F (1999). Proapoptotic Bcl-2 relative Bim required for certain apoptotic responses, leukocyte homeostasis, and to preclude autoimmunity. Science.

[45] Serafin V, Lissandron V, Buldini B, Bresolin S, Paganin M, Grillo F (2017). Phosphoproteomic analysis reveals hyperactivation of mTOR/STAT3 and LCK/Calcineurin axes in pediatric early T-cell precursor ALL. Leukemia.

[46] Kassahn D, Nachbur U, Conus S, Micheau O, Schneider P, Simon H-U (2009). Distinct requirements for activation-induced cell surface expression of preformed Fas/CD95 ligand and cytolytic granule markers in T cells. Cell Death Differ.

[47] Ke H, Harris R, Coloff JL, Jin JY, Leshin B, De Marval PM et al. The c-Jun NH 2 -terminal kinase 2 plays a dominant role in human epidermal neoplasia. Cancer Res. 2010. 10.1158/0008-5472.CAN-09-2923. 10.1158/0008-5472.CAN-09-2923 PMC2855785 20354187

[48] Petronilli V, Penzo D, Scorrano L, Bernardi P, Di Lisa F (2001). The mitochondrial permeability transition, release of cytochrome c and cell death. Correlation with the duration of pore openings in situ. J Biol Chem.

