# Supplementary figures and images for: JNK1 and ERK1/2 modulate lymphocyte homeostasis via BIM and DRP1 upon AICD induction
Source: Cell Death Differ. 2020 Apr 28;27(10):2749–67. doi: 10.1038/s41418-020-0540-1 (PMC7492225; doi:10.1038/s41418-020-0540-1)

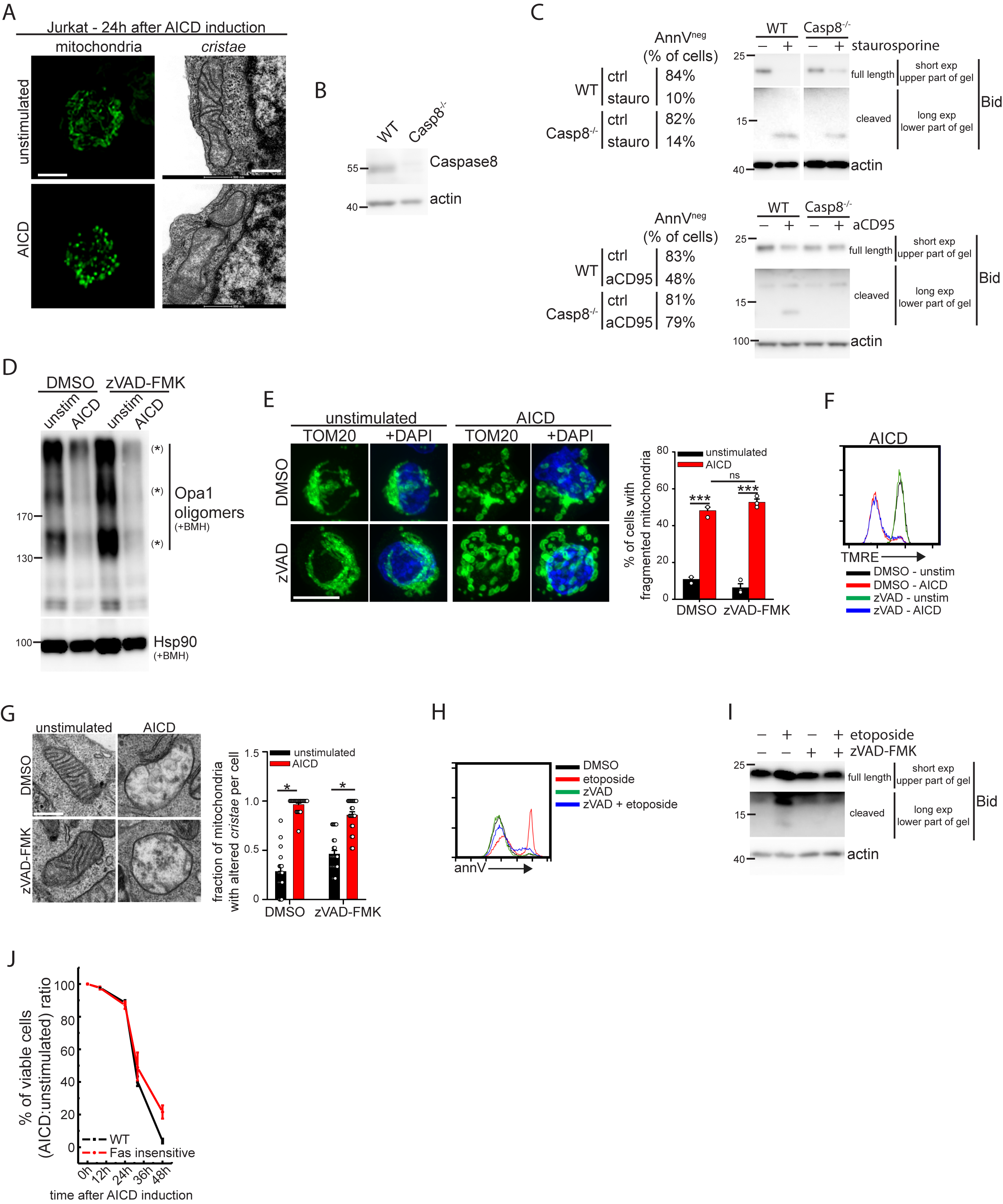

Supplement: Supplementary file 1 — Supplemental Figure 1 [file 41418_2020_540_MOESM1_ESM.tif]

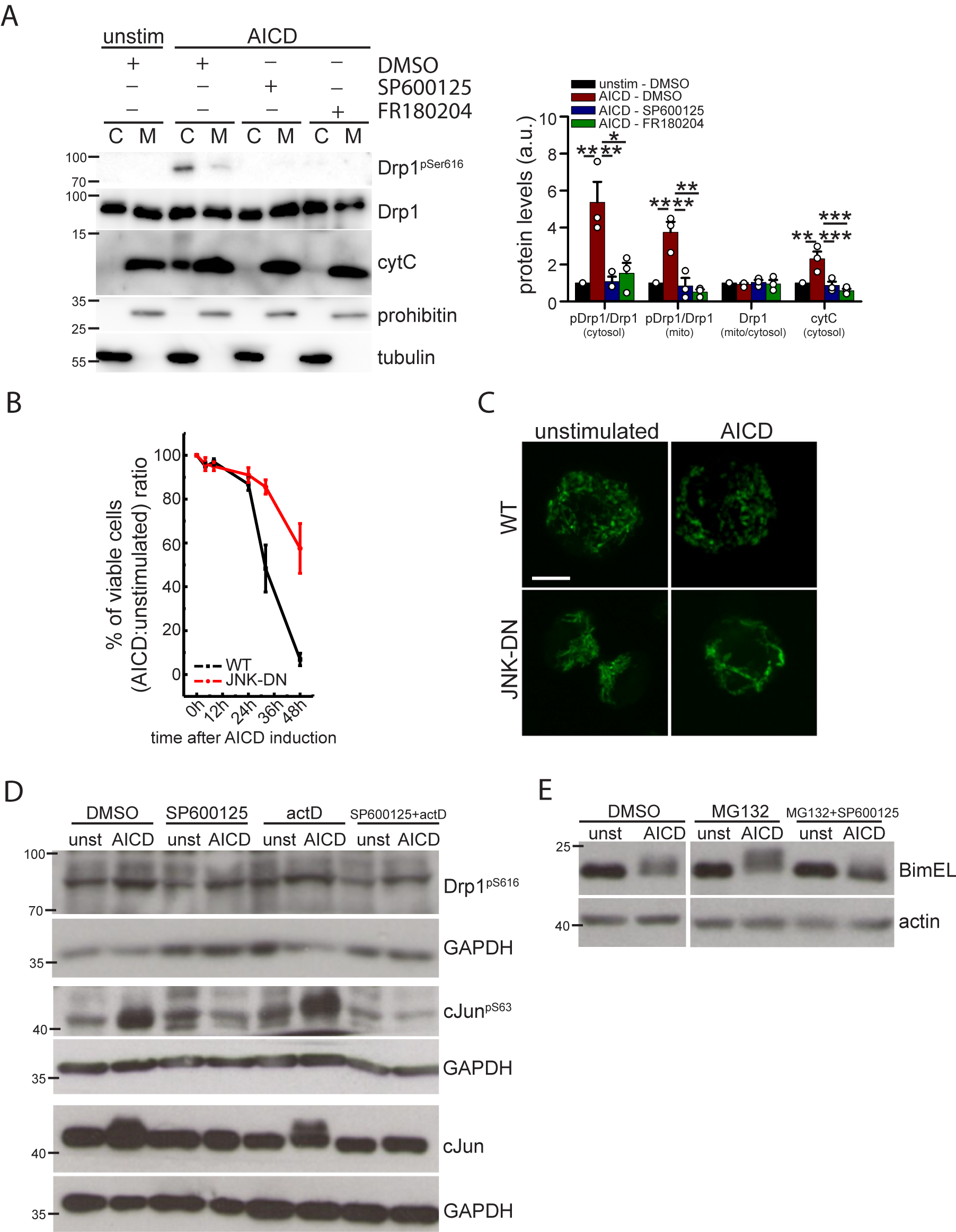

Supplement: Supplementary file 2 — Supplemental Figure 2 [file 41418_2020_540_MOESM2_ESM.tif]

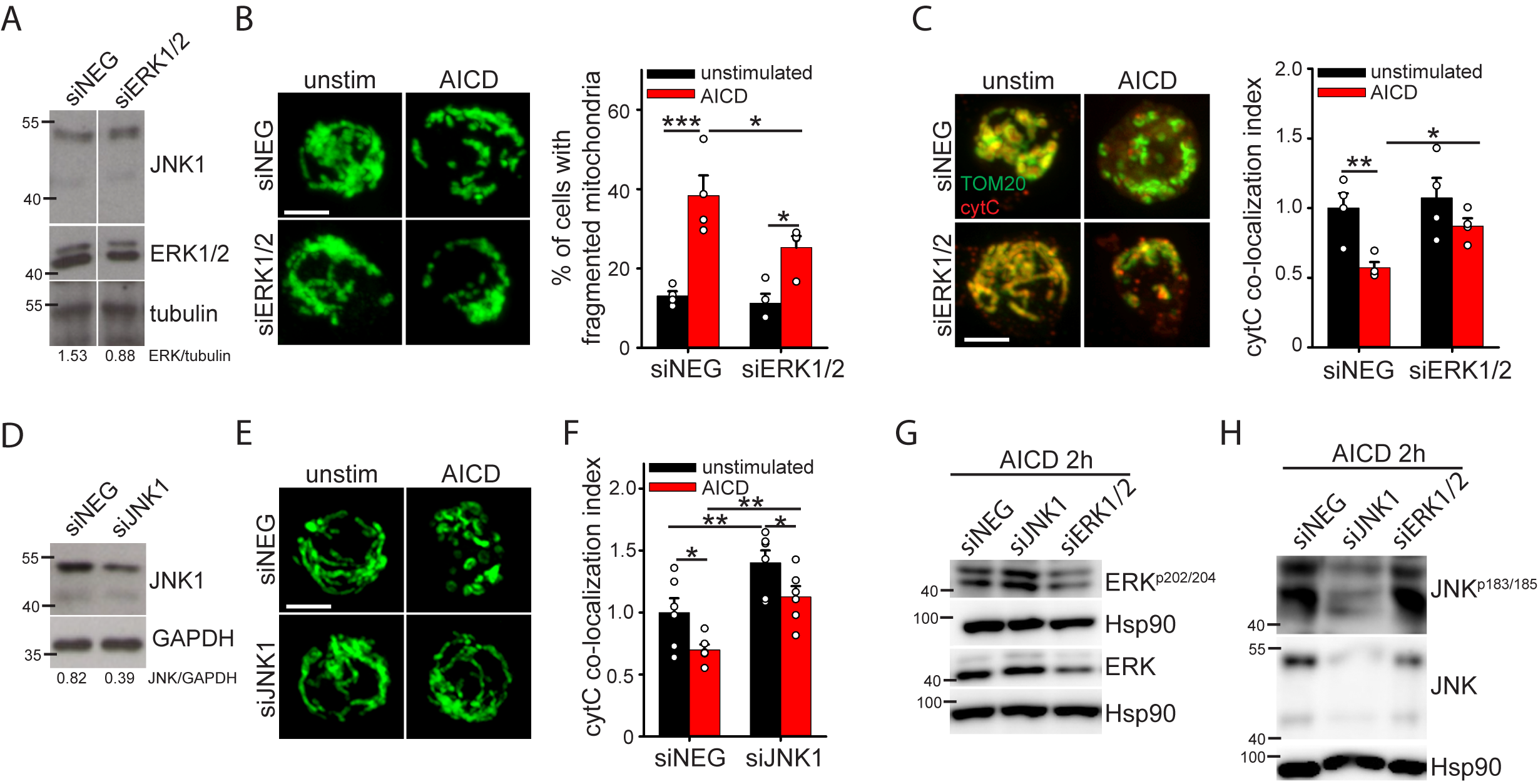

Supplement: Supplementary file 3 — Supplemental Figure 3 [file 41418_2020_540_MOESM3_ESM.tif]

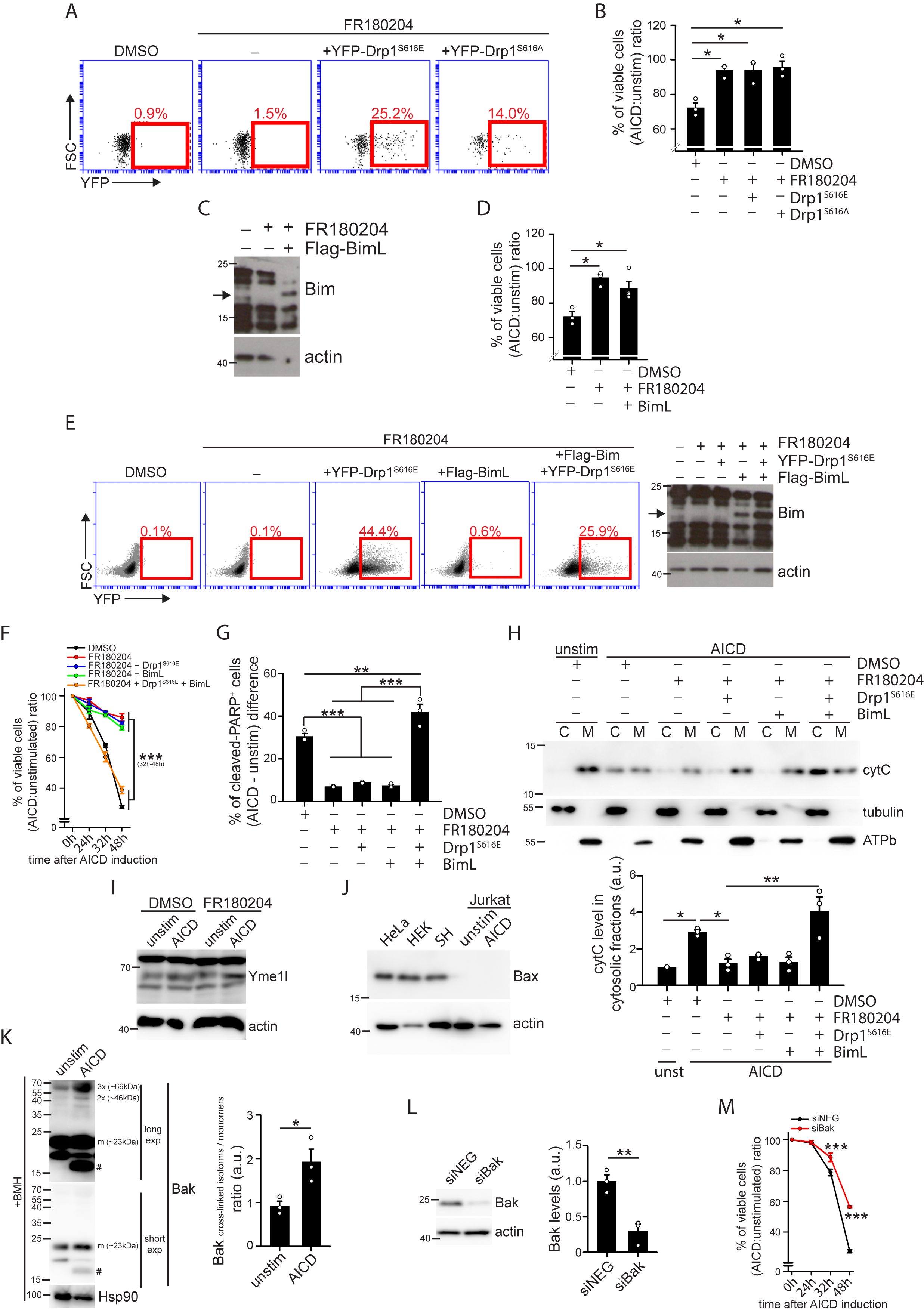

Supplement: Supplementary file 4 — Supplemental Figure 4 [file 41418_2020_540_MOESM4_ESM.tif]

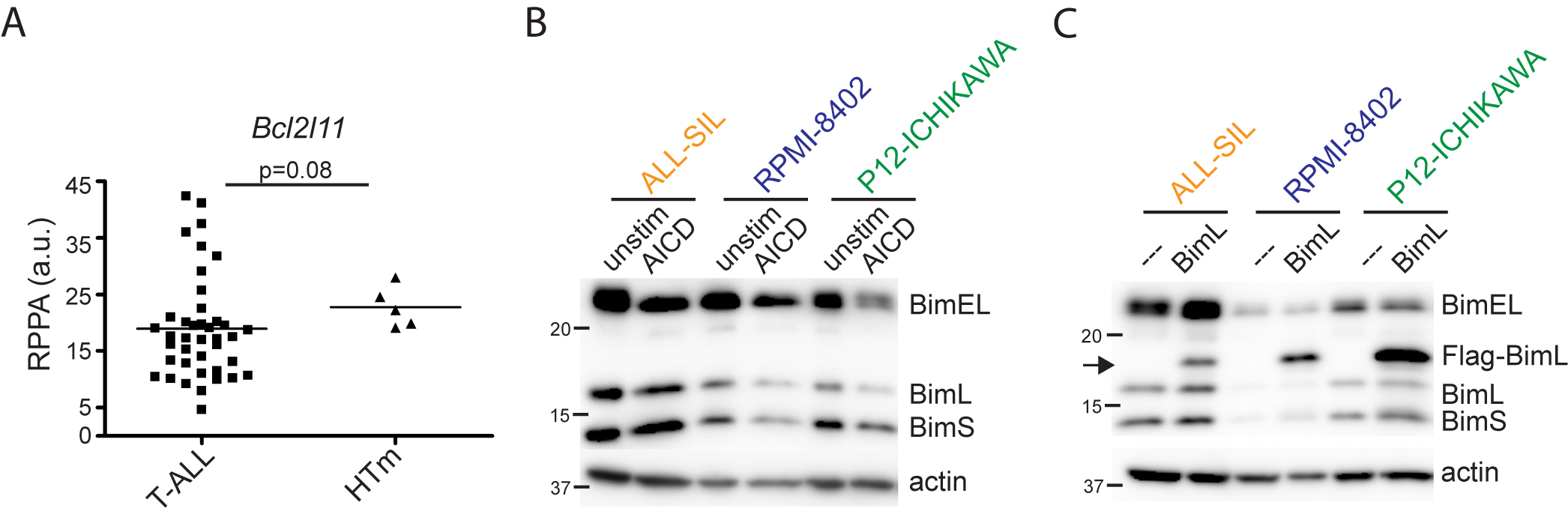

Supplement: Supplementary file 5 — Supplemental Figure 5 [file 41418_2020_540_MOESM5_ESM.tif]

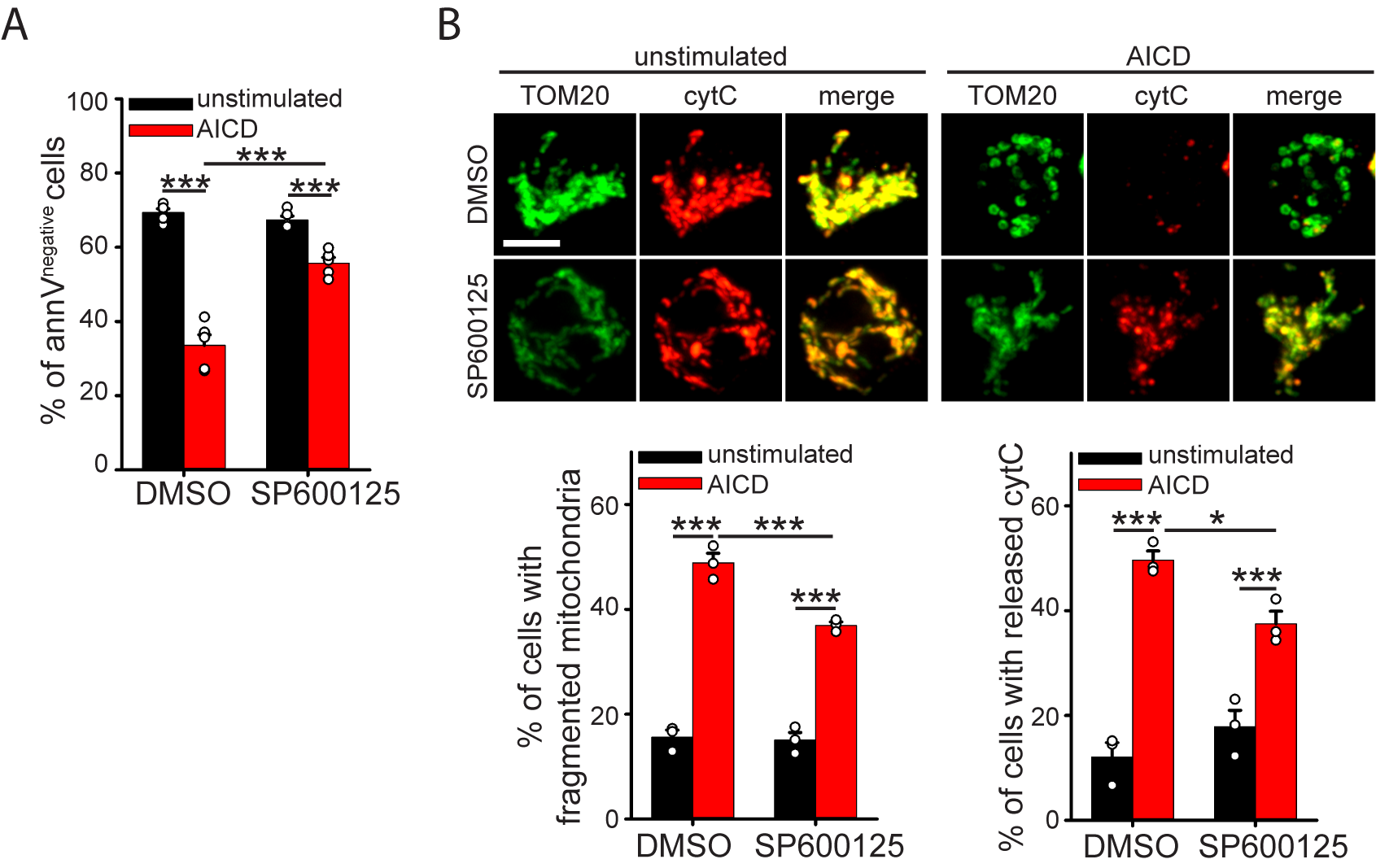

Supplement: Supplementary file 6 — Supplemental Figure 6 [file 41418_2020_540_MOESM6_ESM.tif]
