# Supplementary material for: JNK1 and ERK1/2 modulate lymphocyte homeostasis via BIM and DRP1 upon AICD induction
Source: Cell Death Differ. 2020 Apr 28;27(10):2749–67. doi: 10.1038/s41418-020-0540-1 (PMC7492225; doi:10.1038/s41418-020-0540-1)
Supplement: Supplementary file 7 — Supplemental Figures Legends [file 41418_2020_540_MOESM7_ESM.docx]

**Supplemental Figure 1. Further analyses of the extrinsic death pathway during AICD. Related to Figure 1.
(A)** Jurkat cells transfected with mtYFP, were fixed 24h after AICD induction. Representative reconstructions of z-stacks of the mtYFP fluorescence (left panel) and representative electron micrographs (right panel) in unstimulated and AICD-induced cells. (**B**) Representative caspase-8 expression levels by western blot in WT and caspase-8^-/-^ Jurkat cells. (**C**) WT and caspase-8^-/-^ Jurkat cells have been treated for 4h with staurosporine or anti-CD95 antibody. The percentage of annexinV^negative^ cells measured by flow cytometry is reported on the left for all conditions, while the relative Bid expression levels measured by western blot are reported on the right (one experiment representative of three different experiments). (**D-G**) Jurkat cells have been stimulated to induce AICD in presence or not of 2µM pan-caspase inhibitor z-VAD-FMK and analysed 28h after AICD induction. In (D) is reported the expression level of Opa-1 oligomers (indicated with asterisks) isolated with BMH (one experiment representative of three independent experiments). In (E) is reported a representative confocal z-stack reconstruction of mitochondria morphology (TOM20). The quantification of the percentage of cells with fragmented mitochondria in each condition is reported in the graph (n=3). In (F) is reported the TMRE profile (one experiment representative of three independent experiments). In (G) are reported representative electron micrographs showing *cristae* morphology in each condition. Quantification of the fraction of mitochondria with altered *cristae* per cell (range 0-1) in each condition is reported in the graph on the right (at least 18 cells per condition from n=3 independent experiments). (**H-I**) Jurkat cells have been treated with etoposide for 4h in presence or not of z-VAD-FMK. In (H) is reported a representative flow cytometric plot of the annexinV-FITC staining in each condition. In (I) are reported the expression levels of the indicated proteins. One experiment representative of three independent experiments. (**J**) Relative viability (AICD:unstimulated ratio) of WT and Fas-insensitive Jurkat cells stimulated for AICD induction (percentage of annexinV^negative^7AAD^negative^ cells assessed by flow cytometry) (n=3).
Data are shown as mean ± SEM. Scale bar, 10μm in **A** (left) and **E**, and 0.5μm in **A** (right) and **G**.

**Supplemental Figure 2. Further characterization of JNK and ERK inhibition during AICD. Related to Figure 2.**(**A**) Expression levels of the indicated (phospho)-proteins in cytosolic (C) and mitochondrial (M) fractions obtained from hPBT cells 4h after AICD induction, in presence or not of SP600125 or FR180204. In the graph on the right the following parameters have been measured: relative Drp1^pSer616^/Drp1 ratio in the cytosolic fractions, relative Drp1^pSer616^/Drp1 ratio in the mitochondrial fractions, relative mitochondria/cytosol ratio for Drp1 in each condition, relative amount of cytC in the cytosolic fractions (n=3). (**B**-**C**) Jurkat cells have been transfected with dominant-negative JNK plasmid (DN-JNK) and stimulated for AICD. Relative viability (AICD:untreated cells ratio) of WT and DN-JNK Jurkat cells (percentage of annexinV^negative^ cells assessed by flow cytometry) is reported in (B) (n=3). Representative confocal z-stack reconstructions of mitochondria (TOM20) in WT and DN-JNK Jurkat cells cells 24h after AICD induction is reported in (C). One experiment representative of two independent experiments). (**D-E**) Expression levels of the indicated (phospho)-proteins in hPBT cells 2h after AICD induction, in presence or not of SP600125, actinomycinD (actD) or MG132. One experiment representative of three independent experiments.
Data are shown as mean ± SEM. Scale bar, 10μm in **C**. Significance is indicated as follows: **=p<0.01; **=p<0.01; ***=p<0.001.

**Supplemental Figure 3. ERK1/2 and JNK1 silencing upon AICD in Jurkat and hPBT cells. Related to Figure 3.**(**A**-**F**) hPBT cells have been activated *in vitro* for 24h and expanded for 6 days. Then they have been transfected with the indicated siRNAs (siNEG, siERK1/2 or siJNK1) and, after 24h, stimulated for AICD. In (A) and (D) are reported the representative expression levels of the indicated proteins immediately before AICD induction. Quantifications are reported below the corresponding lanes (one experiment representative of at least three independent experiments). In (B) and (E) representative images of mitochondria morphology (TOM20) 2h after AICD induction are reported. Quantifications of the percentage of cells with fragmented mitochondria in each condition are reported in the graphs on the right (B: n=4; E is one experiment representative of two independent experiments). In (C) and (F) representative images showing mitochondria (TOM20 in green, confocal z-stack reconstructions) and cytochrome-C (red) localization 2h after AICD induction are reported. Quantifications of the cytochrome-C co-localization index with mitochondria (see Methods for details) are reported in the graphs for each condition (C: n=4; F n=6). (**G**-**H**) Jurkat cells have been transfected with the indicated siRNA (siNEG, siERK1/2 or siJNK1). 24h after transfection the cells have been simulated for AICD. The expression levels of the indicated (phospho)-proteins 2h after AICD induction are reported (one experiment representative of three independent experiments).
Data are shown as mean ± SEM. Scale bar, 10μm in **B**, **C** and **E**. Significance is indicated as follows: *=p<0.05; **=p<0.01; ***=p<0.001.

**Supplemental Figure 4. Further analyses of AICD progression following Drp1 and Bim modulation in FR180204-treated Jurkat cells. Related to Figure 4.**(**A**-**B**) Jurkat cells have been transfected with pEYFP-Drp1-pS616E and pEYFP-Drp1-pS616A plasmids and AICD was induced after 24h in presence or not of FR180204. Representative transfection efficiency is reported in (A). In (B) is reported the relative viability (AICD: unstimulated ratio) of transfected Jurkat cells after 32h from AICD induction (percentage of annexinV^negative^7AAD^negative^ cells assessed by flow cytometry). YFP-Drp1 transfected cells have been gated on YFP-positive cells (n=3). (**C**-**D**) Jurkat cells have been transfected with Flag-BimL and AICD was induced after 24h in presence or not of FR180204. Representative western blot analysis to check transfection efficiency is reported in (C), with arrow indicating Flag-BimL. In (D) is reported the relative viability (AICD: unstimulated ratio) of transfected Jurkat cells after 32h from AICD induction (percentage of annexinV^negative^7AAD^negative^ cells assessed by flow cytometry) (n=3). (**E-H**) Jurkat cells have been transfected with pEYFP-Drp1-pS616E and Flag-BimL plasmids and AICD was induced after 24h in presence or not of FR180204. In (E) are reported representative western blot and dot plot analyses to check for transfection efficiency. Arrow indicates exogenous Flag-BimL. YFP-Drp1 transfected cells have been gated on YFP-positive cells to assess viability. In (F) is reported the relative viability (AICD: unstimulated ratio) of transfected Jurkat cells at the indicated time after AICD induction (percentage of annexinV^negative^7AAD^negative^ cells assessed by flow cytometry) (n=3). In (G) is reported the (AICD - unstimulated) difference in the percentage of cells showing cleaved-PARP expression as assessed by flow cytometry 32h after AICD induction (n=3). In (H) are reported the expression levels of the indicated proteins in cytosolic (C) and mitochondrial (M) fractions obtained from transfected Jurkat cells 32h after AICD induction, in presence or not of FR180204. Quantification of the relative amount of cytochrome-C (cytC) in the cytosolic fractions in each condition is reported in the corresponding graph (n=3). (**I**) Expression levels of the indicated proteins in Jurkat cells 28h after AICD induction, in presence or not of FR180204 (one experiment representative of three independent experiments). (**J**) Expression levels of the indicated proteins in HeLa, HEK-293 (HEK), SH-SY5Y (SH) and Jurkat cells (unstimulated or stimulated for 28h to induce AICD). (**K**) Expression levels of the indicated proteins in Jurkat cells 28h after AICD induction with BMH (+BMH) to isolate Bak oligomers. A hash (#) indicates an intra-molecular cross-linked Bak isoform running faster than the monomer and only observed during AICD. The relative ratio of the cross-linked isoforms (including the shorter isoform and the 2x and 3x oligomers) compared to Bak monomers in each condition is reported in the graph (n=3). (**L-M**) Jurkat cells have been transfected with siNEG or siBak siRNAs. After 24h, cells have been stimulated to induce AICD, and then analysed 28h later. In (L) is reported the representative expression levels of the indicate proteins. In (M) is reported the relative viability (AICD: unstimulated ratio) of transfected Jurkat cells at the indicated time after AICD induction (percentage of annexinV^negative^7AAD^negative^ cells assessed by flow cytometry) (n=3).
Data are shown as mean ± SEM. Significance is indicated as follows: *=p<0.05; ***=p<0.001.

**Supplemental Figure 5. Further analyses on T-ALL cells. Related to Figure 5.**(**A**) RRPA analysis of *Bcl2l11* (Bim) expression in T-ALL patients (n=40) and healthy control thymocytes (HTm; n=5). (**B**) Representative western blot of Bim protein levels in ALL-SIL, P12-ICHIKAWA and RPMI-8402 cells, 32h after AICD induction. Quantification of BimL and BimS protein levels is reported in the graph in Fig. 5J. (**C**) Representative western blot of Bim levels in ALL-SIL, P12-ICHIKAWA and RPMI-8402 cells transfected with either pCDNA3 empty vector (--) or with pCDNA3-Flag-BimL (BimL). Arrow indicates exogenous Flag-BimL.
Data are shown as mean ± SEM.

**Supplemental Figure 6. AICD progression in SP600125-treated murine T cells. Related to Figure 6.**(**A**) Relative viability (AICD:unstimulated ratio) of murine T cells stimulated for AICD in presence or not of SP600125 (percentage of annexinV-negative cells assessed by flow cytometry) (n=5). (**B**) Representative confocal z-stack reconstructions of mitochondria (TOM20, in green) and cytochrome-C (in red) in murine T cells 4h after AICD induction in presence or not of SP600125 (n=4). Quantifications of the percentage of cells with fragmented mitochondria, or released cytochrome-C, are reported in the graphs below (n=3).
Data are shown as mean ± SEM. Scale bar, 5μm in **B**. Significance is indicated as follows: *=p<0.05; ***=p<0.001.
